# An enhanced Draco lizard optimizer for accurate parameter extraction of proton exchange membrane fuel cells

**DOI:** 10.1038/s41598-026-57083-3

**Published:** 2026-06-17

**Authors:** Mohammed H. Alqahtani, Ali S. Aljumah, Ahmed R. Ginidi, Abdullah M. Shaheen

**Affiliations:** 1 Department of Electrical Engineering, College of Engineering, Prince Sattam bin Abdulaziz University, Al Kharj, 16278 Saudi Arabia; 2 Department of Electrical Engineering, Faculty of Engineering, Suez University, P.O. Box: 43221, Suez, Egypt

**Keywords:** PEM Fuel Cell, Modified Draco Lizard Optimizer, Parameter Extraction, Fuel Cell Modeling, Energy science and technology, Engineering, Mathematics and computing

## Abstract

Accurate parameter extraction is crucial for the modelling of proton exchange membrane (PEM) fuel cells, which involves complex, non-linear, and multivariate relationships essential for simulation, design, and fault diagnostics. This paper proposes a Modified version of the Draco Lizard Optimizer (MDLO) technique to precisely extract important PEM fuel cell parameters. This hybridization aims to increase optimization efficiency by striking a balance between exploration and exploitation. The efficacy of MDLO is supported by extensive simulations that use three commercially available PEM fuel cell systems to compare its performance to that of the conventional DLO and new metaheuristic optimization approaches, which are Driving Training-Based Optimization (DTBO), Moss Growth Optimization, and Skill Optimization Algorithm (SOA). Best fitness, average fitness, worst fitness, standard deviation, convergence speed, and multiple-comparison test are among the performance indicators that are applied and measured during the course of 55 runs. According to the findings, MDLO provides the best Sum of Squared Errors (SSE) value, greater accuracy, dependability, speed of convergence, and a strong fit for the estimated primary parameters. The runs’ low and consistent SSE values—0.331348 for the 250 W, 1.1698 $\:\times\:$ 10^− 2^ for the BCS 500 W, and 2.100246 for the NedStack PS6—provide effectiveness and robustness of the MDLO.

## Introduction

Accurate parameter estimation for proton exchange membrane fuel cells (PEMFCs) is essential for developing reliable mathematical models that support simulation, design, control, and optimization in clean energy applications. The PEMFC model is highly nonlinear and multivariable, with unknown parameters (such as activation voltage coefficients, ohmic resistance, and mass transport coefficients) that must be identified from experimental voltage-current (V-I) polarization data. Traditional gradient-based methods often struggle with the non-convex, multimodal nature of the objective function—typically formulated as the minimization of the sum of squared errors (SSE) between measured and model-predicted voltages—leading to the widespread adoption of metaheuristic optimization algorithms^1,2^.

Recent years have witnessed a surge in the application of bio-inspired and physics-inspired metaheuristic algorithms for PEMFC parameter extraction. These methods offer gradient-free exploration, robustness to local optima, and adaptability without requiring differentiability of the objective function. Researchers have benchmarked them against standard test cases, such as Ballard Mark V, BCS 500-W, and NedStack PS6 stacks, using metrics like SSE, convergence speed, and statistical consistency across multiple runs^3^. Bio-inspired swarm and evolutionary optimizers have formed a foundational approach such as an enhanced bald eagle search algorithm that was applied to PEMFC model optimization^4^. Building on this trajectory, a Walrus optimizer inspired by walrus foraging and migration behaviors was introduced in^5^ achieving competitive Sum of Squared Error (SSE) values and stable performance in extracting PEMFC parameters. In^6^, the rime-ice algorithm, which simulated rime ice formation processes, was evaluated reporting an SSE of approximately 1.945 on benchmark stacks and superior convergence compared to moth-flame optimizer, Grey Wolf Optimizer (GWO), and other contemporaries. In^3^, a Hippopotamus Optimization Algorithm (HOA) was modified incorporating different exploitation mechanisms and an enhanced solution quality strategy. This approach effectively mitigated premature convergence issues common in the base HOA and outperformed GWO, and chimp optimization algorithm in PEMFC variable extraction.

Physics-inspired algorithms have also gained traction. In^7^, Young’s Double-Slit Experiment (YDSE or YSDE) algorithm was developed, drawing from wave interference principles in classical physics, and applied to PEMFC mathematical modeling and parameter estimation. Additionally, in^8^, a reinforcement learning-based technique was presented for PEMFC parameter estimation, leveraging learning from interaction with the environment to adaptively tune parameters and potentially offering advantages in dynamic or online scenarios over purely evolutionary methods. Complementary studies extend these insights. Nevertheless, Bakır and Ağbulut (2026) addressed efficient design, operation, and control of commercial PEMFCs in^9^, emphasizing the role of accurate parameter identification in practical deployment. Bakır (2026)^10^ further explored state-of-the-art metaheuristics in related photovoltaic models, highlighting transferable techniques for energy system parameter extraction. Also, several optimizers have been revealed to handle the PEMFC parameter estimation challenge: shark smell optimizer (SSO)^11^, slime mould optimizer (SMO)^12^, manta rays foraging optimizer (MRFO)^13^, black widow optimizer (BWO)^14^, chaotic Harris hawk optimizer (CHHO)^15^, bonobo optimizer (BO)^16^, pathfinder optimizer (PFO)^17^, tree-growth optimizer (TGO)^18^, jellyfish search optimizer (JSO)^19^, coyote optimizer (CO)^20^, flower pollination optimizer (FPO)^21^, improved artificial ecosystem optimizer (IAEO)^22^.

In^23^, a unique Parrot optimisation approach based on the flexible eating, communication, and fear-responsive behaviours of Pyrrhura Molinae parrots has been created and used to PEMFC parameter optimisation. The approach incorporated Lévy flight processes to optimise the proportion of both discovery and extraction, and the SSE was minimised for various PEMFC stacks, indicating increased estimate accuracy. In^24^, an improved hierarchical population-based differential evolution method was established, in which unconventional diversity measures, such as mean distances, mean standard deviations, and mean squared distances, were integrated to continuously adjust the trade-off between discovering and exploiting in PEMFC optimisation problems. This dynamic diversity controlling technique has proven to increase convergence behaviour and solution reliability. In^25^, a hybridised slime mould- improved converging particle swarm optimiser has been implemented. This technique incorporated numerous upgrade mechanisms, such as excellent point set initialisation, a detection attacking strategy developed from coati optimisation, and a sigmoid-driven nonlinear scaled process. These modifications are intended to expedite convergence and improve global searching capacity throughout PEMFC parameter determination. In^26^, an improved artificially generated hummingbird approach (EAHA) has been constructed to find the seven undetermined variables within PEMFC assemblies. The suggested EAHA improved the effectiveness of searching by combining various territorial forage techniques with a linear controlling function. The efficiency of the traditional AHA was contrasted with that of the EAHA among three frequently used PEMFC modules, and enhanced convergence accuracy as well as resilience were found. In^27^, a Rüppell’s Fox Optimiser (RFO) based on Rüppell’s fox perceptual hunt behaviour has been designed for optimising the Mann PEMFC model’s seven variables. The method has been verified using three distinct PEMFC stacks, including Horizon H-12, Ballard Mark V, and Temasek 1 kW. The SSE has been successfully reduced, resulting in steady convergence with precise I-V and I-P feature fitting under a variety of operating situations. In addition, Sobol global sensitivity analysis has been included, allowing for parameter effect evaluation and model simplification.

Metaheuristic optimization algorithms, inspired by natural phenomena and biological behaviors, are vital for solving complex non-linear and high-dimensional optimization problems in various domains like engineering and energy systems. Notable methods, including Particle Swarm Optimization (PSO), Genetic Algorithms (GA), and Differential Evolution (DE), provide flexibility and are effective when traditional methods fail due to their computational challenges or constraints^28^. Classical metaheuristics, despite their advantages, face issues such as premature convergence to local optima, poor balance between exploration and exploitation, sensitivity to parameter settings, and reduced effectiveness on specific problems like constrained optimal power flow, parameter identification, and multi-objective dispatch. These challenges stem from their generic design, which often fails to preserve population diversity, adaptively manage promising solutions, or effectively navigate complex search spaces filled with non-linearities and high dimensionality^29^.

Modifications to classical algorithms are crucial for improving convergence speed, solution quality, robustness, and domain-specific applicability. Key operators targeted include selection, mutation/reproduction, and exploration/exploitation mechanisms. Common techniques include Lévy flights for exploration, chaotic maps for randomness, opposition-based learning, adaptive parameter control, and various mutation operators. The Fitness-Distance Balance (FDB) selection method, including its dynamic and roulette variants, has gained traction by selecting guided solutions that balance fitness quality and proximity, thereby reducing premature convergence. Recent studies illustrate these trends. In^30^, the Lévy Flight Distribution algorithm with an FDB-based guiding mechanism was improved for AVR system design, demonstrating superior performance through better search agent selection. Subsequent works extended FDB to Chimp optimization, adaptive gaining-sharing knowledge algorithms, and artificial rabbits optimization, yielding enhanced results in global optimization and power system stabilizer/OPF problems^31,32^. Dynamic FDB variants and switched crowding mechanisms have further advanced multi-objective PSO applications in AC-DC OPF. In^33^, a mutated trochoid searching optimizer was introduced for combined heat and power dispatch with reserves. Additionally, an adaptive fitness-guided Starfish optimizer was introduced for power flow optimization^34^ and secure reconfigurable interface surfaces communication^35^. In both investigations, a fitness-based interaction mechanism was incorporated into the exploitation phase of the Starfish optimiser, which was inspired by the biology of starfish. Without requiring extra control settings, this improvement lowered the danger of premature convergence, preserved population variety, and increased the accuracy of local searches. In^36^, an improved Elk Herd algorithm was introduced where a new best-bull-guided differential reproduction mechanism has been added to the Elk Herd method. By exploiting scaled differences between randomly chosen herd members to disturb the global best solution, this improvement enabled the generation of a percentage of offspring solutions. Consequently, the process preserved population variety and delays premature convergence while strengthening exploitation in attractive regions. Gafar et al. (2026) applied novel fuzzy-enhanced Pied Kingfisher Optimization (PKO) strategy^37^ for wireless network optimization. In this work, energy-based motion control, adaptive subgrouping, flock cooperation, and memory-driven re-perching were incorporated in the classic PKO Algorithm. Inspired by human search and rescue operations, the search and rescue algorithm was applied in^38^ to the OPF problem. In this technique, scattered search agents explored the solution space broadly to locate potential victims in the search phase while focused efforts concentrated around found solutions for refinement in the rescue phase. In^39^, the snow ablation optimizer was implemented for the economic dispatch. In this work, the natural processes of snow direct transition from solid to gas, melting, and evaporation were modelled. These models were divided into widespread particle dispersion during sublimation/evaporation as exploration strategy, and localized refinement as snow melts and concentrates as exploitation strategy. In^40^, a hybrid Artificial Protozoa Optimizer (APO)–PSO metaheuristic was developed for diagnosing and monitoring intelligent transformers. This approach integrated PSO’s efficient social information-sharing mechanisms with APO’s adaptable biological search strategies, which enhanced solution diversity and mitigates premature convergence. In^41^, a modified Fire Hawk Optimizer (FHO) was developed to emulate the foraging behavior of fire hawks, aimed at extracting solar cell model parameters. The FHO includes enhancements to the phasor operator with phase angles, transforming it into a trigonometric nonparametric, self-adaptive balanced algorithm, while integrating a dimension learning-based hunting strategy, which resulted in reduced diversity.

Moreover, two improvements to the Artificial Hummingbird Algorithm (AHA) are covered in^42,43^. The first improvement, in^42^, used a dynamic FDB operator, to boost the AHA’s search capabilities. By varying its weight coefficient during the optimisation process, this operator enabled exact balance of exploration and exploitation. The Natural Survivor Method (NSM-AHA), which substituted the traditional greedy survivor selection with a novel mechanism based on NSM, is the second innovation mentioned in^43^. Both a fitness score and an adaptation score obtained from the relationships within the population were used by this process to evaluate individuals. In^44–46^, various algorithms utilizing a dynamic variant of the FDB mechanism were developed to address optimal power flow challenges in power systems. Ref^44^. introduced a dynamic FDB-based artificial rabbits algorithm aimed at optimizing power flow. Ref^45^. focused on an improved adaptive gaining-sharing knowledge algorithm that integrated an FDB-based approach for reactive power flow management, particularly in networks with distributed generation and High Voltage Direct Current (HVDC) links. Additionally, Ref^46^. described enhancements to a Phasor PSO method by incorporating FDB to tackle optimal power flow issues within hybrid AC/DC grids that employed Voltage Source Converter (VSC)-based multi-terminal HVDC links. The implementation of the FDB selection mechanism contributed to a better balance between exploration and exploitation within the complex search landscape.

These studies reveal that targeted modifications in algorithms, such as FDB-based selection and adaptive reproduction, improve performance significantly. They lead to faster convergence, enhanced robustness, better constraint satisfaction, and higher solution quality in various fields, including energy systems and engineering design. The evidence supporting algorithm hybridization and operator refinement encourages ongoing innovation in modified metaheuristics for tackling complex optimization challenges.

Although self-adaptive nature optimizers are useful for predicting the unknown values for PEMFCs, they still need to be improved in areas including statistical analysis, time burden, and convergence speed. As therefore, the Draco Lizard Optimizer (DLO), which was first presented in^47^, is a novel metaheuristic optimization algorithm that draws inspiration from the distinctive gliding, navigation, camouflage, and adaptive maneuvering capabilities of the Draco lizard. In order to improve the efficiency of classification, the DLO was also utilized for feature selection when combined with Support Vector Machines, demonstrating its efficacy and dependability in feature selection tasks^48^.

In order to identify PEM fuel cell parameters, this study proposes a Modified Draco Lizard Optimizer (MDLO). In order to improve adaptability, stability, and biological realism, the proposed MDLO incorporates three significant improvements to the current DLO. Initially, the original algorithm’s strict phase division is replaced with a fuzzy Gaussian-based model to control exploration and exploitation, guaranteeing more seamless behavioral changes. Second, a continuous exponential steering vector decaying is suggested, which permits gradual modulation of search intensity and removes the binary steering mechanism’s discontinuities. Third, a random boundary re-initialization strategy is presented to improve solution variety and avoid early stagnation brought on by boundary clustering. This article’s main novelties are:

- A modified DLO algorithm is developed using a fuzzy Gaussian-based model, a random boundary re-initialization method and a continuous exponential steering vector decaying.
- The modified MDLO for the NedStack PS6 PEM Fuel Cell, the BCS 500 W, and the 250 W is presented through experimental validation.
- The outcomes of the proposed MDLO are compared with the conventional DLO and recently developed techniques which are Driving Training-Based Optimization (DTBO)^49^; Moss Growth Optimization (Moss)^50^; Skill Optimization Algorithm (SOA)^51^.
- The acquired findings validate the superiority of the MDLO over the conventional DLO and recently developed techniques .
- The MDLO’s resilience is demonstrated by statistical research on fuel cell models.

The rest of the sections are organized as follows: Section II offers a desirable formulation of the PEMFC dynamic paradigm. Section III clarifies the conventional DLO and the proposed modified DLO for PEMFC, whereas the simulation outcomes of the MDLO and recently developed techniques are displayed in Section IV, when employed on the three PEMFC stacks. Section V outlines the investigation’s main findings.

## Problem formulation: mathematical modelling of PEMFC

**Fig. 1:**
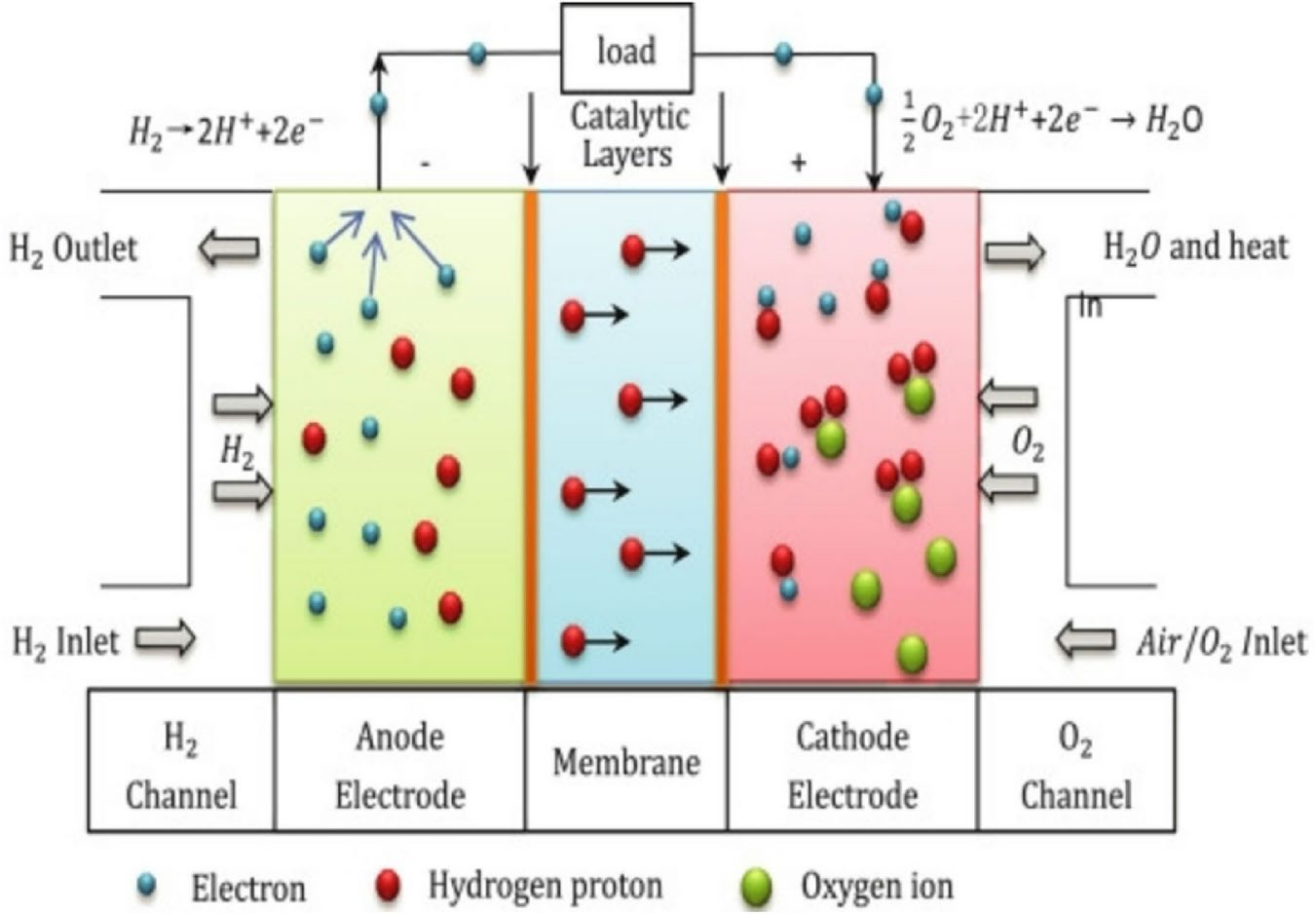
Schematic of the PEMFC^52^.

A PEMFC system comprises two electrodes and an electrolyte, as demonstrated in Fig. 1. The electrolyte is distinguished by its capacity to block electrons while allowing positive ions (protons) to pass through. At one electrode, referred to as the anode, hydrogen gas is injected and, with the aid of a catalyst, divided into electrons and hydrogen protons. The following is a summary of the particular responses involved:

1 $$H_{2} \to 2H^{ + } + 2e^{ - }$$

2 $$\frac{1}{2}O_{2} + 2H^{ + } + 2e^{ - } \to H_{2} O$$

3 $$H_{2} + \frac{1}{2}O_{2} \to H_{2} O + Electric\,current + Heat$$

### Model of PEMFC

The electrochemical simplified model suggested by Mann et al.^53^ is used in this article to illustrate the PEMFC’s steady state performance. The PEMFC model can be created by analytically displaying the I-V characteristics (Polarization curves). Due to inadequate reaction rate, insufficient reactant gas transport, and fuel cell electrical resistance, the actual PEMFCs output voltage is lower than the desired voltage. Therefore, the real output voltage (*V*_*Cell*_)^54^ can be written as follows:

4 $$V_{{Cell}} = N_{{cells}} .(E_{{Nrnst}} - v_{{ohm}} - v_{{act}} - v_{{con}} )$$

where $E_{{Nrnst}}$represents the reversible equilibrium voltage and *N*_*cells*_ *is* series-connected cells, while *V*_*ohm*_, *V*_*act*_, and $v_{{con}}$represent the ohmic voltage drop, the activation overpotential, and the concentration overpotential, respectively. Equation (2) can be used to determine the voltage at a reference temperature of 25 °C. These three voltage drop quantities are therefore given as shown in Eqs. (5)–(7)^55^. The potential of the cell acquired in an open-circuit is known as the $E_{{Nrnst}}$, and it can be computed using the $E_{{Nrnst}}$Equation as follows^56^:

5 $$E_{{Nernst}} = - 0.85(T_{{fc}} - 298.15)/1000 + 4.3085/10^{5} \times T_{{fc}} \ln (P_{{H_{2} }} \sqrt {P_{{O_{2} }} } ) + 1.229$$

6 $$v_{{act}} = - \left[ {\xi _{1} + T_{{fc}} (\xi _{2} + \xi _{3} \ln (C_{{O_{2} }} ) + \xi _{4} \ln (I_{{fc}} ))} \right]$$

Where $$C_{{O_{2} }} = \frac{{P_{{O_{2} }} }}{{5.08 \times 10^{6} }}.\exp (\frac{{498}}{{T_{{fc}} }})$$

7 $$v_{{ohm}} = I_{{fc}} (R_{m} + R_{c} );R_{m} = \rho _{m} l/M_{A}$$

Where $$\rho _{m} = \frac{{181.6\left[ {1 + 0.03I_{{fc}} /M_{A} + 0.062\left( {T_{{fc}} /303} \right)^{2} \left( {I_{{fc}} /M_{A} } \right)^{{2.5}} } \right]}}{{\left[ {\lambda - 0.634 - 3I_{{fc}} /M_{A} } \right].\exp \left[ {4.18 \times \left( {(T_{{fc}} - 303)/T_{{fc}} } \right)} \right]}}$$

8 $$v_{{con}} = - \beta .\ln \left( {1 - J/J_{{\max }} } \right)$$

where $C_{{O2}}$ demonstrates the oxygen concentration (mol/cm^3^),$I_{{fc}}$illustrates the operating current (*A*), whilst$P_{{O2}}$and $P_{{H2}}$ explain the partial pressures of oxygen (atm) and hydrogen, respectively. Moreover,$T_{{fc}}$ characterizes the FC working temperature (*K*). Besides, *ξ*_*1*_ − *ξ*_4_ exemplify parametric coefficients, *M*_*A*_ indicates the membrane area (*cm*)^55,57^.

In addition to this, *R*_*c*_ and *R*_*m*_ expose the electron-transfer and the membrane (Ω) ohmic resistances, respectively, whereas *l* is the membrane thickness (cm). Furthermore, the symbol ($\rho _{m}$) establishes the resistivity for the electron flow (Ω.cm), whilst *λ* and *β* are changeable parameters within an empirical range. and whilst *J*_*max*_ and *J* (with (*A/cm*^*2*^)) designate the maximum and actual thermal current densities, respectively^58^.

9 $$\beta = \wp .T_{{fc}} /2\alpha F$$

where $\wp$, *F* and characterize ideal gas and Faraday constants, respectively, whereas the symbol *α* illustrates charge transfer coefficient. A further examination of Eqs. (5) and (6) reveals that the concentration voltage drop can be modulated in a linear relationship with temperature and real current density. For instance, the concentration of polarization voltage is anticipated to grow due to increased cell temperatures and greater current densities. In order to create a suitable PEMFC model for any upcoming power system study, it may be inferred that the seven parameters are essentially estimated.

### Problem formulation and description

Establishing an objective function of FC (FCG) is crucial in order to use optimization techniques to find the ideal values of the seven unknown factors previously discussed. The goal function in this study is the sum of the squared errors (SSE) resulting from the PEMFC stack’s actual output voltage and the output voltage estimated by the model that is illustrated as follows^59,60^:

10 $$Min(FCG) = \left( {\sum\limits_{{z = 1}}^{{N_{S} }} {\left[ {V_{{FC,\mathrm{ex} }} (z) - V_{{FC,es}} (z)} \right]^{2} } } \right)$$

where *N*_*S*_ illustrates the number of experimental data points, *z* demonstrates the iteration counter, $V_{{FC,es}}$characterizes the output voltage of the model, and *V*_*FC, ex*_ characterizes the measured output voltage. The unknown parameters vector are {*R*_*c*_, *λ*, *β*, and *ξ*_*1*_ to *ξ*_*4*_} that can support to obtain the best value of the SSE by using the proposed MDLO. For the unknown parameters, this FCG is limited by inequality constraints that represent the minimum and maximum limits of these parameters.

## Proposed MDLO for PEM fuel cell parameter estimation

### Conventional Draco Lizard Optimizer (DLO)

The two main stages of the Draco Lizard Optimizer are modeled after the mobility and survival strategies of Draco lizards. Later generations of the algorithm focus on exploitation through concealment and adaptive maneuvering, whereas earlier rounds prioritize exploration through gliding and navigation methods. The optimization process continues until the predetermined maximum number of iterations )*IT*_*max*_( is reached by applying a greedy selection technique at each iteration to retain superior solutions.

#### Step 1: Initialization

In order to prepare for exploration, Draco lizards disperse throughout a new environment during the initialization phase. In the search space, each lizard’s position (*Dr*_*i*_) is represented as a vector, where Dim stands for the number of optimization variables. The population is expressed mathematically as follows and has *N*_*Dr*_ potential solutions:

11 $$Dr = {\text{ }}\left[ {Dr_{1} ,Dr_{2} ,Dr_{3} ,.........Dr_{{N_{{Dr}} }} } \right]^{T}$$

Every potential solution (*Dr*_*i*_) is characterized by: with each individual defined as:

12 $$Dr_{i} {\text{ }} = {\text{ }}\left[ {Dr_{{i,1}} ,Dr_{{i,2}} ,Dr_{{i,3}} ,.........Dr_{{i,Dim}} } \right];{\text{ i}} = 1:N_{{Dr}}$$

Each location vector’s components are created at random within the permitted search space to reflect the inherent unpredictability in choosing perches or branches:

13 $$Dr_{{i,k}} {\text{ }} = {\text{ }}Lp\_Dr_{k} {\text{ }} + \overrightarrow {{R_{1} }} .\left( {Up\_Dr_{k} {\text{ }} - {\text{ }}Lp\_Dr_{k} } \right);{\text{ i}} = 1:N_{{Dr}} ;{\text{ k}} = 1:Dim$$

The k-th decision variable’s lower and upper limits are represented by *Lp_Dr*_*k*_ and *Up_Dr*_*k*_, respectively, whereas $\:\overrightarrow{{R}_{1}}$ is a uniformly distributed arbitrary vector in the interval [0,1]. Similar to how Draco lizards occupy accessible and secure areas within their habitat, this formulation guarantees that all initial solutions fall into the acceptable domain. Following initialization, the objective function is used to assess each candidate solution and determine its fitness value (*FCG*_*i*_). The swarm is guided during successive iterations by the global best (*Dr*_*B*_), which is the solution with the lowest fitness value (*FCG*_*B*_).

#### Step 2: DLO Gliding and Navigation

Draco lizards use their exceptional gliding skills to move between trees, avoid predators, and find food in the natural world. The lizard perceives impediments, modifies its flight route, and lands perfectly on a far-off tree during its meticulously planned glide, which is not haphazard. Similar to a lizard navigating new perches without duplicating previous positions, the DLO directs searching agents to new solutions while incorporating prior best placements. This phase, which focuses on global exploration, mathematically controls the first half of the optimization process (*it*<*ITmax*/2). The following rule governs how each candidate’s solution modifies its position:

14 $$Dr_{i}^{{it + 1}} = \left\{ {\begin{array}{*{20}c} {Dr_{B} - r_{a} \times \left( {\left( {A \times Dr_{B} } \right) - Dr_{m}^{{it}} } \right) + \overrightarrow {{DL}} \times r_{a} \times \left( {Dr_{B} - Dr_{m}^{{it}} } \right)} & {if{\text{ }}FCG_{B} < FCG_{m} } \\ {Dr_{B} + r_{a} \times \left( {Dr_{B} - Dr_{m}^{{it}} } \right) + \overrightarrow {{DL}} \times r_{a} \times \left( {Dr_{B} - Dr_{m}^{{it}} } \right)} & {Else} \\ \end{array} } \right.$$

where two randomly chosen individuals’ positions, $\:{Dr}_{m}^{it}$ and $\:{Dr}_{n}^{it}$, represent various trees the lizard sees while in flight. The symbol (*A*) is a rounded integer in {1,2} that occasionally permits stronger directional movements, indicating abrupt gliding alterations. Similar to the manner in which a Draco lizard deliberately modifies its wing angles and tail to maneuver in midair, *DL* is a dynamic steering vector that controls which dimensions of the solution space are altered at each step:

15 $$DL = \left\{ {\begin{array}{*{20}c} 1 \\ {P_{1} \left( k \right)} \\ {P_{2} \left( k \right)} \\ {P_{3} \left( k \right)} \\ 0 \\ \end{array} } \right.{\text{ }}\begin{array}{*{20}c} {it < \phi _{1} \times IT_{{\max }} } \\ {it < \phi _{2} \times IT_{{\max }} } \\ {it < \phi _{3} \times IT_{{\max }} } \\ {it < \phi _{4} \times IT_{{\max }} } \\ {it < \phi _{5} \times IT_{{\max }} } \\ \end{array}$$

where pattern vectors are illustrated by *P*_*1*_= [0,1,1,1], *P*_*2*_= [0,0,1,1], *P*_*3*_= [0,0,0,1] and parameter sets are denoted by *ϕ*_*1*_ = 0.1, *ϕ*_*2*_ = 0.2, *ϕ*_*3*_ = 0.3, *ϕ*_*4*_ = 0.4, *ϕ*_*5*_ = 0.5.

#### Step 3: Camouflage and adaptive maneuvering in DLO

A Draco lizard exhibits two amazing survival strategies—camouflage and adaptable maneuvering—when confronted with predators or difficult environments. In order to evade notice, the lizard occasionally stays almost immobile, fitting in perfectly with the dead leaves or bark. In other situations, it moves quickly and nimbly to reposition itself and get away from danger. During the second part of the iteration phase (*it*≥ *IT*_*max*_/2), the DLO initiates this step. In order to avoid being stuck in local optima, the DLO alternates between local fine-tuning and sporadic long jumps, moving from wide exploration to concentrated refining.

16 $$Dr_{i}^{{it + 1}} = \left\{ {\begin{array}{*{20}c} {Dr_{B} + Levy\left( {Dim} \right) \times \left( {Dr_{B} - Dr_{l}^{{it}} } \right)} & {if{\text{ }}r_{b} < \rho } \\ {Dr_{B} + g \times \left( {\frac{{IT_{{\max }} - it}}{{IT_{{\max }} }}} \right) \times \left( {Dr_{B} - Dr_{l}^{{it}} } \right)} & {Else} \\ \end{array} } \right.$$

where *g* adds Gaussian noise in order to permit tiny local mutations, *ρ* = 0.2 denotes the probability of employing a Lévy flight step (escape strategy), and $\:{Dr}_{l}^{it}$ indicates a randomly selected person that represents environmental choices.

#### Greedy selection

The boundary limits are assessed to ensure every variable is still inside the potential search range, following each of Draco’s seeking solutions has been upgraded. By projecting any excessive values back into permitted bounds, this technique avoids out-of-range solutions:

17 $$Dr_{{i,k}}^{{it + 1}} = {\text{ }}\min \left( {\max \left( {Dr_{{i,k}}^{{it + 1}} ,Lp\_Dr_{k} } \right),Up\_Dr_{k} } \right);{\text{ i}} = 1:N_{{Dr}} ;{\text{ k}} = 1:Dim$$

DLO assesses each potential solution by contrasting it with the individual’s best location. The historical best is adjusted as follows if the new position results in a lower fitness value:

18 $$Dr_{i}^{{it + 1}} = \left\{ {\begin{array}{*{20}c} {Dr_{i}^{{it + 1}} } \\ {Dr_{i}^{{it}} } \\ \end{array} } \right.{\text{ }}\begin{array}{*{20}c} {if{\text{ }}FCG_{i}^{{it + 1}} \le FCG_{i}^{{it}} } \\ {Otherwise} \\ \end{array} ;{\text{ i}} = 1:N_{{Dr}}$$

Furthermore, the global best gets revised in the following manner if the candidate offers a more suitable solution than the existing one:

19 $$Dr_{B} = \left\{ {\begin{array}{*{20}c} {Dr_{i}^{{it + 1}} } \\ {Dr_{B} } \\ \end{array} } \right.{\text{ }}\begin{array}{*{20}c} {if{\text{ }}FCG_{i}^{{it + 1}} \le FCG_{B} } \\ {Otherwise} \\ \end{array} ;{\text{ i}} = 1:N_{{Dr}}$$

**Fig. 2:**
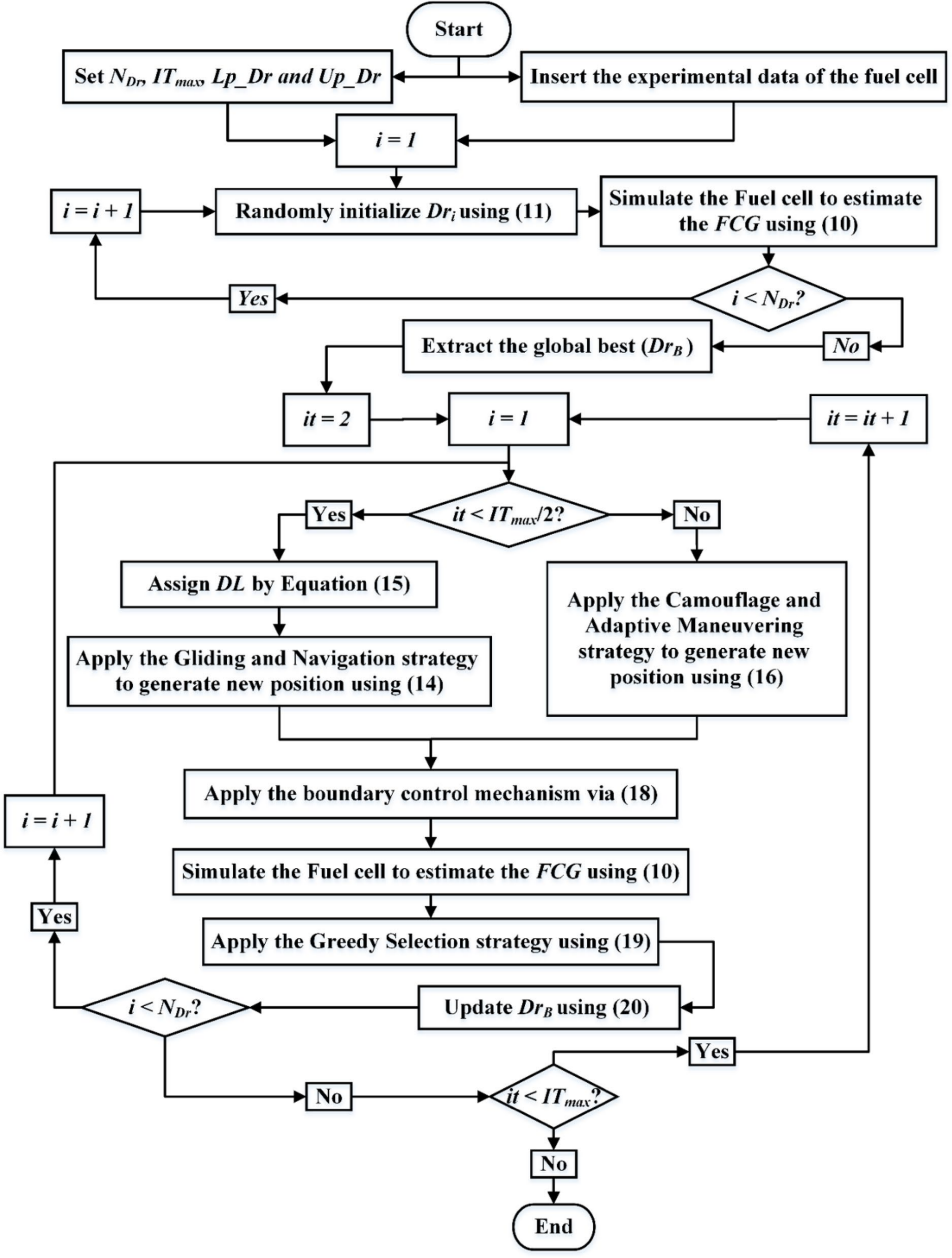
Main steps of DLO.

Thus, by encouraging survival and resource optimization, supporting greedy selection, and fostering monotonic enhancements to solution effectiveness, DLO guarantees a progressive, consistent evolving process. The DLO’s primary steps can be seen in Fig. 2.

### Proposed MDLO with Fuzzy Phase Control and Adaptive Steering Mechanism

Three significant improvements are intended to increase adaptability, reliability, and realistic biology in the suggested modified version of the DLO (MDLO). Initially, the conventional technique’s stiff half-split framework is replaced with a fuzzy Gaussian-based exploitation–exploration controller. This prevents premature convergence whilst preserving adaptability by enabling a seamless, probabilistic transition among exploitation and exploration across iterations. Second, a continuous exponentially decreasing function that progressively lessens the strength of positional shifts over time has taken the place of the piecewise steering controlling system (Sigma). This guarantees that agents emulate the Draco lizard’s gliding changes as it reaches a desired location by making larger exploration movements early on and gradually smaller improvements afterward. at last, an agent’s choice between exploration and exploitation is determined.

The proposed MDLO is theoretically grounded in addressing the fundamental exploration–exploitation trade-off that governs metaheuristic convergence behavior. In the original DLO, the deterministic half-iteration phase switching introduces a discontinuous transition in search dynamics, which may lead to premature convergence. By incorporating a fuzzy Gaussian-based control mechanism, the MDLO establishes a probabilistic and continuous transition between exploration and exploitation phases. This soft switching preserves population diversity in early iterations while ensuring gradual intensification around high-quality regions as iterations progress. Furthermore, replacing the binary steering vector with a continuous exponentially decaying function creates smooth behavior. Initial iterations enable significant positional updates for broader search space coverage, while later iterations focus on smaller adjustments for improved localization. This approach prevents abrupt changes that could destabilize convergence, facilitating consistent progression towards stable optimal solutions. Additionally, the proposed random boundary re-initialization strategy enhances the algorithm’s diversity preservation by uniformly redistributing violated components within feasible bounds, unlike the original DLO’s boundary clipping, which limits exploration by concentrating agents near search-space borders. These three modifications enhance stability, convergence smoothness, and robustness without increasing algorithmic complexity. The MDLO therefore maintains the biological inspiration of DLO while embedding principled stochastic control and adaptive step-size mechanisms that align with established metaheuristic optimization theory.

#### Exploration/exploitation control using fuzzy model

A fixed iteration level (e.g., *it* < *IT*_*max*_/2 for exploration, else exploitation) separated exploration from exploitation in the conventional DLO. Search behavior may oscillate or prematurely converge as a result of this sudden shift. To enable both exploration and exploitation to exist simultaneously in each iteration, a fuzzy transition mechanism is added in the improved version. Gaussian membership functions are used to dynamically calculate the probability of choosing each option:

20 $$\mu _{{\exp lore}}^{{it}} = \exp \left( {\frac{{ - \left( {it - {\raise0.7ex\hbox{${IT_{{\max }} }$} \!\mathord{\left/ {\vphantom {{IT_{{\max }} } 4}}\right.\kern-\nulldelimiterspace} \!\lower0.7ex\hbox{$4$}}} \right)^{2} }}{{2 \times \left( {{\raise0.7ex\hbox{${IT_{{\max }} }$} \!\mathord{\left/ {\vphantom {{IT_{{\max }} } 4}}\right.\kern-\nulldelimiterspace} \!\lower0.7ex\hbox{$4$}}} \right)^{2} }}} \right)$$

21 $$\mu _{{\exp loit}}^{{it}} = \exp \left( {\frac{{ - \left( {it - 3 \times {\raise0.7ex\hbox{${IT_{{\max }} }$} \!\mathord{\left/ {\vphantom {{IT_{{\max }} } 4}}\right.\kern-\nulldelimiterspace} \!\lower0.7ex\hbox{$4$}}} \right)^{2} }}{{2 \times \left( {{\raise0.7ex\hbox{${IT_{{\max }} }$} \!\mathord{\left/ {\vphantom {{IT_{{\max }} } 4}}\right.\kern-\nulldelimiterspace} \!\lower0.7ex\hbox{$4$}}} \right)^{2} }}} \right)$$

where *IT*_*max*_ represents the highest number of iterations, and the symbol (*it*)denotes the current iteration. The following is the normalized likelihood of exploration:

22 $$\rho _{{\exp lore}}^{{it}} = \frac{{\mu _{{\exp lore}}^{{it}} }}{{\mu _{{\exp lore}}^{{it}} + \mu _{{\exp loit}}^{{it}} }}$$

where the normalized probability for exploration is represented by $\:{\rho\:}_{{exp}lre}^{it}$. Based on these probabilities, the suggested MDLO selects exploration or exploitation at each iteration:

23 $$Mode_{i}^{{it}} = \left\{ {\begin{array}{*{20}c} {Exploration} \\ {Explotation} \\ \end{array} } \right.{\text{ }}\begin{array}{*{20}c} {if{\text{ r}}_{b} {\text{ < }}\rho _{{\exp lore}}^{{it}} } \\ {Else} \\ \end{array}$$

where *r*_*b*_ is a uniformly distributed random number. This keeps variability during the search and avoids premature stagnation by ensuring a hazy overlap between exploration and exploitation.

To further clarify the adaptive transition mechanism in Eq. (23), the proposed MDLO does not employ a rigid deterministic switching rule between exploration and exploitation phases. Instead, the transition is governed through a continuous probabilistic regulation mechanism controlled by the fuzzy Gaussian transition coefficient, which varies adaptively with the iteration index. The purpose of this formulation is to gradually shift the search behavior from exploration-dominant movement during the early optimization stages toward exploitation-oriented refinement during the later iterations.

At the beginning of the optimization process $\:\left(it≪I{T}_{max}\right)$, the value of $\:{\mu\:}_{{exp}lore\:}^{it}$remains relatively high, resulting in a larger probability of activating exploration operators. In this stage, candidate solutions are encouraged to perform wider search-space traversal, stochastic directional movement, and diversity-preserving updates in order to avoid premature convergence and improve global search coverage. As the iteration count increases and approaches the maximum iteration number, the value of $\:{\mu\:}_{e{xp}lore\:}^{it}\:$gradually decreases due to the exponential decay mechanism, thereby increasing the probability of exploitation-oriented updates. Consequently, the algorithm progressively emphasizes local refinement around promising regions and improves convergence precision.

Unlike the original DLO, which applies abrupt half-iteration switching between exploration and exploitation, the proposed MDLO introduces a smooth and continuous transition process. This adaptive behavior prevents sudden search instability and allows the optimizer to maintain balanced exploration capability while simultaneously strengthening convergence accuracy during later iterations.

For illustrative clarification, assume that $\:I{T}_{max}=1000$. During the early stage at iteration $\:it=100$, the computed transition coefficient may remain relatively high (e.g., $\:{\mu\:}_{{exp}lore}^{it}=0.835$), indicating that exploration operators dominate the update mechanism. At the middle stage $\:\left(it=500\right)$, the transition coefficient decreases (e.g., $\:{\mu\:}_{{exp}lore}^{it}=0.6$), producing a balanced exploration–exploitation behavior. Finally, near the termination stage $\:\left(it=900\right)$, the coefficient becomes small (e.g., $\:{\mu\:}_{{exp}lore}^{it}=0.034$), causing exploitation-oriented local search and convergence refinement to dominate the optimization process. This gradual transition mechanism enables MDLO to preserve population diversity during early iterations while ensuring stable convergence and accurate parameter extraction during the final optimization stages.

#### Adaptive continuous steering vector decay

The steering vector $\:\overrightarrow{DL}\:$stated in Eq. (15) in the conventional DLO is binary, accepting values {0,1}, with abrupt changes every predetermined number of repetitions. Discontinuities and a lack of control over the intensity of exploitation resulted from this. The steering vector $\:\overrightarrow{DL}\:$described in the proposed MDLO is represented by a continuous, exponentially decaying function that smoothly ranges from 1 (high unpredictability during the initial iterations) to 0.5 (refined search in late iterations):

24 $$\mathop {DL}\limits^{ \to } = \frac{1}{4} + \frac{1}{4}\exp \left( { - \lambda \times \frac{{it}}{{IT_{{\max }} }}} \right)$$

where the declining amplitude is denoted by λ. Large exploratory movements across several dimensions are made possible by this model’s initial dominance of the exponential term. The exponential term progressively decreases towards a minimum threshold of 0.25 as the number of iterations increases, forcing smaller, more precise motions in subsequent iterations. in contrast to the conventional DLO’s sudden limit adjustments. By preventing abrupt behavioral changes that could result in oscillations in the level of quality of the solution, this approach enhances the consistency of the search procedure.

#### Boundary control approach via re-initialization

In order to address boundary violations, the preliminary DLO framework used Eq. (17) to trim values which were out of boundaries to the closest border. This frequently resulted in a loss of diversity and increased search difficulty. A developed variant employs a random re-initialization strategy, which implies it examines the violated dimension uniformly within the permitted range rather than instantly assigning the variable to the border:

25 $$\begin{gathered} Dr_{{i,k}}^{{it + 1}} = {\text{ }}\left\{ {\begin{array}{*{20}c} {Dr_{{i,k}}^{{it + 1}} } \\ {{\text{ }}Lp\_Dr_{k} {\text{ }} + \overrightarrow {{R_{1} }} .\left( {Up\_Dr_{k} {\text{ }} - {\text{ }}Lp\_Dr_{k} } \right)} \\ \end{array} } \right.{\text{ }}\begin{array}{*{20}c} {Up\_Dr_{k} \ge {\text{ }}Dr_{{i,k}}^{{it + 1}} \ge {\text{ }}Lp\_Dr_{k} } \\ {Else} \\ \end{array} \hfill \\ {\text{ i}} = 1:N_{{Dr}} ;{\text{ k}} = 1:Dim \hfill \\ \end{gathered}$$

**Fig. 3:**
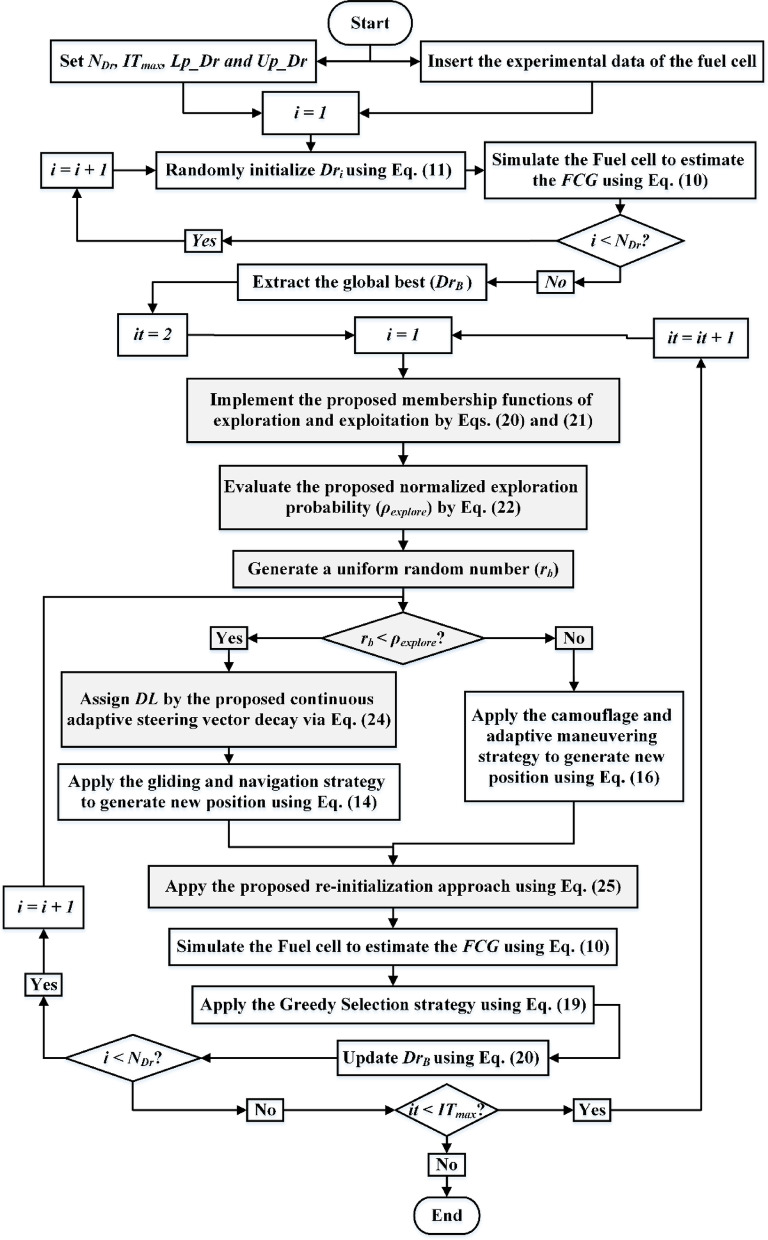
Main steps of the proposed MDLO.

As updated alternatives are redistributed within the search scope, the method ensures that every changed variable falls inside the viable search domain, maintaining population diversity and improving search efficiency by removing premature standstill caused by boundary clustering. The key steps of the proposed new DLO version are depicted in Fig. 3.

To ensure reproducibility, the pseudo-code of the proposed MDLO is illustrated in Algorithm 1. To address statistical fairness, random seed control was applied during all experimental evaluations. For deterministic replication, a fixed seed value was set prior to execution. For robustness analysis, 55 independent runs were conducted using distinct seed values. This approach guarantees unbiased stochastic sampling while preserving repeatability of reported results.

**Algorithm 1:**
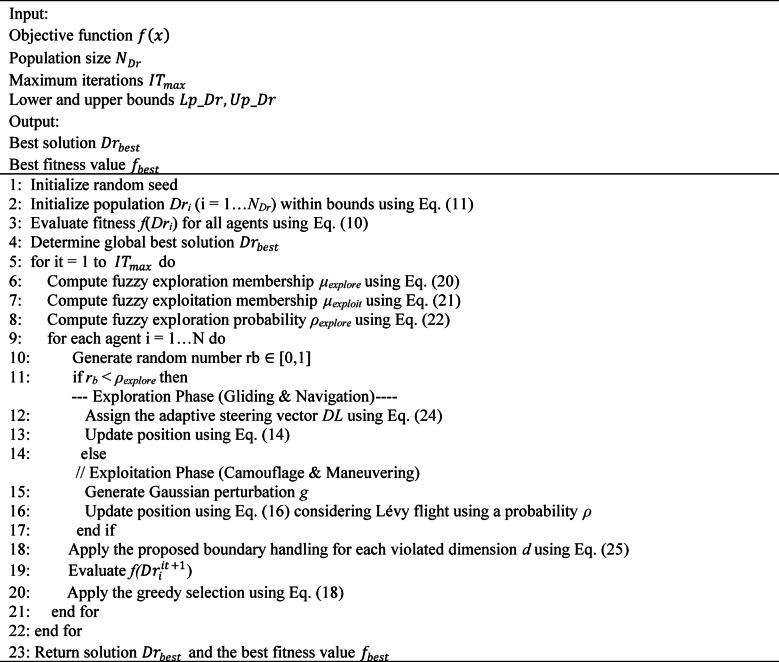
Proposed MDLO.

#### Fundamental novelty of the proposed MDLO

While many recent metaheuristic improvements rely primarily on parameter tuning, adaptive coefficient adjustment, or hybridization with external algorithms, the proposed MDLO introduces a structural reformulation of the internal search dynamics of the original DLO rather than merely modifying numerical constants. The theoretical novelty of MDLO lies in three fundamental aspects: (i) replacing deterministic phase partitioning with a probabilistic fuzzy-transition framework, thereby transforming the search process from a rigid two-stage mechanism into a continuously adaptive stochastic system; (ii) reformulating the discrete steering vector into a continuously decaying control law that mathematically embeds a time-varying step-size adaptation analogous to annealing-based convergence schemes; and (iii) redefining boundary handling from a corrective projection operator to a diversity-preserving stochastic redistribution operator.

Unlike common hybrid metaheuristics that integrate external search operators (e.g., PSO, DE, or Lévy mutations) into a base algorithm, The MDLO therefore maintains the biological inspiration of DLO while embedding principled stochastic control and adaptive step-size mechanisms that align with established metaheuristic optimization theory. Consequently, the novelty is not confined to parameter calibration or superficial structural extension but instead lies in redefining the exploration–exploitation regulation model, step-size dynamics, and boundary feasibility strategy at the algorithmic control level. This positions MDLO as a control-theoretic refinement of DLO rather than a conventional hybrid variant.

## Results and discussion

This study demonstrates the performance of the suggested MDLO to acquire FC parameter extraction using three examples of common commercial PEMFC stacks: BCS 500 W, 250 W, and NedStack PS6. Table 1 Practical boundaries and datasheets for the unidentified parameters of commercial PEMFCs BCS 500 W, 250 W, and NedStack PS6. Moreover, the fitness function is defined as SSE to minimize the total squared difference between experimental and computed voltages under a set of practical restrictions.

To ensure a rigorous and unbiased comparative assessment, all implemented algorithms, including MDLO, conventional DLO, DTBO, Moss, and SOA, were executed under identical computational conditions. Each algorithm was initialized with a population size of 30 search agents and operated under the same stopping criteria, defined as a maximum of 300 iterations and an equivalent limit of 90,000 objective function evaluations. These standardized control parameters ensure fairness, and methodological consistency across all comparative evaluations.

To mitigate concerns of overfitting from low SSE values and small standard deviations, the semi-empirical Mann model is clarified to include only seven meaningful parameters related to activation, ohmic, and concentration losses. These parameters are confined to realistic electrochemical ranges, minimizing over-parameterization and avoiding arbitrary curve fitting. Therefore, the optimization process adjusts physically interpretable quantities rather than flexible black-box coefficients. Moreover, robustness was confirmed through 55 independent runs and variance analysis, showing consistent convergence behavior and algorithmic stability, as indicated by small dispersion in the proposed MDLO. Validation across three PEMFC stacks with varying power ratings and datasets reinforces generalizability. The method’s stable performance under real conditions, despite noise and fluctuations, demonstrates its reliable predictive capability across diverse operating scenarios.

**Table 1:** Datasheets with the practical limits for the unknown parameters for NedStack PS6, BCS 500 W, and 250 W.

Stack type	Technical Specifications			Practical Boundaries			
	NedStack PS6	BCS 500 W	250 W	Parameter		Min	Max
*T*_*c*_ *(K)*	343	333	343	*λ*		10	23
*P*_*O2*_ *(atm)*	1	0.2095	1	*β(V)*		0.0136	0.5
*L (µm)*	178	178	127	*ξ*_*2*_ *0.10* ^*− 3*^ *(V/K)*		1	5
*P*_*H2*_ *(atm)*	1	1	1.000	*R*_*c*_ *(mΩ)*		0.1	0.8
*A*_*m*_ *(cm* ^*2*^ *)*	240	64.0	24.0	*ξ*_*3*_ *0.10* ^*− 5*^ *(V/K)*		3.6	9.8
*J*_*m*_ *(A/cm* ^*2*^ *)*	1.4	0.469	0.860	*ξ*_*4*_ *0.10* ^*− 5*^ *(V/K)*		-26	-9.54
*N*	65	32	24	*ξ*_*1*_ *(V)*		-1.1997	-0.85320

The absolute values of the obtained SSE differ across the three PEMFC stacks (BCS 500 W, 250 W, and NedStack PS6). This variation is expected and does not necessarily reflect differences in optimization quality, but rather arises from intrinsic differences in stack ratings, current ranges, voltage magnitudes, and the number of experimental data points used in each dataset. Since the SSE is scale-dependent, larger stacks with higher voltage and power ranges naturally produce higher absolute error magnitudes. Therefore, direct comparison of SSE values across different fuel cell models is not meaningful in absolute terms. Instead, performance consistency should be interpreted through relative metrics such as percentage voltage absolute error (VAE), percentage power absolute error (PAE), convergence stability, and statistical dispersion across independent runs. From a sensitivity perspective, the semi-empirical Mann model involves seven coupled nonlinear parameters whose sensitivity is influenced by operating conditions such as temperature, membrane area, and current density. These factors alter the optimization landscape and parameter interactions. The consistent convergence of MDLO across stacks ranging from 250 W to 6 kW demonstrates robustness against model-scale variations and parameter coupling effects. Hence, the algorithm’s performance is not stack-specific but generalizes effectively across different PEMFC sizes and operating conditions.

### BCS 500 W PEMFC stacks

This PEM stack, which has a maximum current of 30 A and a rated power of 500 W, is primarily produced by the American company BCS Technologies^61^. The proposed MDLO is revealed to identify the ideal stack settings that produce the best PEMFC model values, as demonstrated in Table 2. As manifested in this table, the MDLO attained a small value of SSE of 1.1698$\:\times\:$10^-2,^ which is lower than the conventional DLO, SOA, Moss, and DTBO, which attain SSE values of 1.5469$\:\times\:$10^-2^, 1.1821$\:\times\:$10^-2^, 1.2175$\:\times\:$10^-2,^ and 4.0323$\:\times\:$10^-2^, respectively. Furthermore, to exemplify the MDLO robustness, Table 2 characterizes the comparison between new reported techniques and the MDLO. The reported techniques are vortex search algorithm (VSA)^62^, grasshopper optimization algorithm (GOA)^22^, an improved algorithm based on the Heap-based optimizer IHBO^63^, Harris Hawks’ optimization (HHO)^64^, ant lion optimizer (ALO)^22^, firefly optimization algorithm (FOA)^57^, moth- fame optimizer (MFO)^65^, whale optimization algorithm (WOA)^66^, equilibrium optimizer (EO)^63^, shuffled frog-leaping algorithm (SFLA)^57^, sine tree-seed algorithm (STSA)^63^, salp swarm algorithm (SSA)^67^, multi-verse optimizer (MVO)^22^, fractional-order modified HHO (FMHHO)^68^, manta rays foraging optimizer (MRFO)^63^, Salp Swarm Optimizer (SSO)^60^, atom search optimization (ASO) techniques^64^, imperialist competitive algorithm (ICA)^57^, modified HHO (MHHO)^68^, and VSA with differential evolution (VSDE)^62^. In terms of SSE, it is evident that the MDLO surpasses the conventional DLO and both the published technique and the new techniques in this study.

**Table 2:** Parameters extracted from the BCS500W using MDLO compared with DLO and reported techniques.

Technique	ξ_1_ (V)	ξ_2_$\:\times\:$10^−4^(V/K)	ξ_2_$\:\times\:$10^−5^(V/K)	ξ_2_$\:\times\:$10^−4^(V/K)	λ	*R*_c_(mΩ)	β(V) $\:\times\:$10^− 2^	SSE $\:\times\:$ 10^− 2^
MDLO	-1.198837	37.3812	6.95889	-1.93023	20.88169455	0.10000	1.61285	1.1698
DLO	-0.903027	30.2737	8.11947	-1.91722	21.75236211	0.268417	1.49313	1.5469
SOA	-0.950958	25.4612	4.11358	-1.76601	14.66958677	0.100876	1.36000	1.1821
Moss	-0.860005	25.5483	5.90993	-1.92228	21.67693957	0.128791	1.63553	1.2175
DTBO	-0.895103	24.3336	4.42684	-1.92456	23	0.312179	1.61154	4.0323
IHBO[63]	-1.19970	33.100	4.2000	-1.9300	20.877	0.100	1.613	1.170
HHO [69]	-0.96053	33.505	8.7377	-1.8967	21.821	0.42358	1.5006	1.5753
FOA[65]	-1.035664	29.502	3.7669451	-0.9540000	15.029691	0.1622	1.3600	1.1819
SSA[68]	-1.0074	33.470	8.1500	-1.9000	18.9165	0.121	1.50	1.610
ASO[64]	-1.0432	36.745	8.8772	1.8775	23.3295	0.581379	1.6495	2.661
ICA [65]	-1.034322	33.202	6.4420795	-0.9540000	15.09701	0.165	1.3600	1.1856
FMHHO[69]	-0.87884	30.236	8.2272	-1.1934	22.709	0.40472	1.5289	1.1770
GOA[22]	-0.8550	30.320	9.0600	-1.9000	21.0423	0.319	1.46	1.710
VSDE[62]	-1.1970	42.330	9.7990	-1.9201	20.194	0.1108	1.57	1.214
WOA[67]	-1.19693	31.800	3.6000	-1.7700	22.974	0.100	2.216	37.273
VSA[62]	-1.0005	3.0053	5.8273	-1.9498	22.322	0.2161	1.58	1.570
MRFO[63]	-1.11262	30.600	4.2300	-1.9500	21.705	0.111	1.718	3.683
STSA[63]	-0.85320	21.800	3.8300	-1.9100	18.062	0.100	1.383	2.135
SFLA[65]	-0.965740	34.760	7.7883354	-0.9540000	15.03229	0.162	1.3600	1.1697
SSO[60]	-0.9719	33.487	7.9111	-0.95435	13.0000	0.10000	5.34	1.219
MHHO[69]	-0.91048	30.661	7.9053	-1.9098	19.384	0.10320	1.5212	1.3511
MFO[66]	-1.0079	33.230	7.9800	-1.9000	20.9189	0.154	1.58	1.190
ALO[22]	-1.1880	36.840	6.8200	-1.9000	22.5552	0.290	1.60	1.190
HHO[64]	-1.09311	32.8141	5.67397	-1.89666	20.0436	0.225793	151.48	1.4879
MVO[22]	-1.1396	31.910	4.5800	-1.9000	20.5547	0.410	1.41	2.130

Table 3 presents a statistical comparison of various methods to assess the effectiveness of the suggested MDLO. The effectiveness of the suggested MDLO in comparison to other revolutionary optimizers for calculating the parameters of the BCS500W fuel cell stack is displayed in Table 3. The techniques are GOA^22^, AEO^69^, ALO^22^, FOA^57^, WOA^66^, MFO^65^, MVO^22^, MHHO^68^, SSO^60^, EO^63^, MRFO^63^, SSA^67^, STSA^63^, ASO^64^, IHBO^63^, HHO^64^, FMHHO^68^, and HHO^68^. The statistical results presented in Table 3 clearly demonstrate the superiority of the proposed MDLO for the BCS500W stack. MDLO achieves the lowest best value of $\:1.1698\times\:{10}^{-2}$and the smallest mean value of $\:1.1706\times\:{10}^{-2}$among the conventional DLO and all compared optimizers, indicating high solution accuracy. Moreover, MDLO exhibits an extremely low standard deviation of $\:2.33\times\:{10}^{-5}$, which reflects excellent robustness and stable convergence behavior over repeated runs. In contrast, most reported optimizers show noticeably higher mean and worst-case values, accompanied by significantly larger standard deviations, revealing sensitivity to initial conditions and premature convergence. Although a few algorithms such as IHBO and SOA provide competitive best values, their statistical dispersion remains higher than that of MDLO. These results confirm that the proposed MDLO offers a reliable and efficient performance for the BCS500W stack.

**Table 3:** Statistical comparison of the MDLO with various methods for the BCS500W Stack.

Technique	Best ($\:\times\:$10^− 2^)	Mean ($\:\times\:$10^− 2^)	Worst ($\:\times\:$10^− 2^)	STD
MDLO	1.1698	1.1706	1.1811	2.33$\:\times\:$*10*^*−5*^
DLO	1.5469	4.8958	13.7143	2.3634$\:\times\:$*10*^*−2*^
SOA	1.1821	1.3523	2.4081	2.518$\:\times\:$*10*^*−3*^
Moss	1.2175	1.7633	3.1542	4.774$\:\times\:$*10*^*−3*^
DTBO	4.0323	4.75086	153.6896	3.6733$\:\times\:$*10*^*−1*^
MRFO [63]	3.683	39.107	113.428	2.5386$\:\times\:$*10*^*−1*^
SSA [68]	1.61	16.23	47.80	1.565$\:\times\:$*10*^*−1*^
IHBO [63]	1.170	1.174	1.185	6 $\:\times\:$ *10*^*− 5*^
WOA [67]	37.273	256.370	851.732	2.55770
FOA [65]	1.1819	-	-	-
MFO [66]	1.19	4.78	13.51	4.34$\:\times\:$*10*^*−2*^
HHO [69]	1.5753	28.200	42.233	1.3165$\:\times\:$*10*^*−1*^
SSO [60]	1.219	-	2.25060	2.03 $\:\times\:$*10*^*− 2*^
HHO [64]	1.4879	-	-	-
FMHHO [69]	1.770	37.371	13.498	1.3008 $\:\times\:$*10*^*− 2*^
ALO [22]	1.19	20.60	60.52	1.880 $\:\times\:$*10*^*− 1*^
GOA [22]	1.71	43.8549	221.0571	67.4693
ASO [64]	2.661	-	-	-
MVO [22]	2.13	5.25	13.56	1.565 $\:\times\:$*10*^*− 1*^
MHHO [69]	1.3511	24.067	55.450	1.5958 $\:\times\:$*10*^*− 1*^
STSA [63]	2.135	62.114	340.177	7.0331$\:\times\:$*10*^*−1*^
EO [63]	1.462	4.646	13.414	3.633$\:\times\:$*10*^*−2*^

**Fig. 4:**
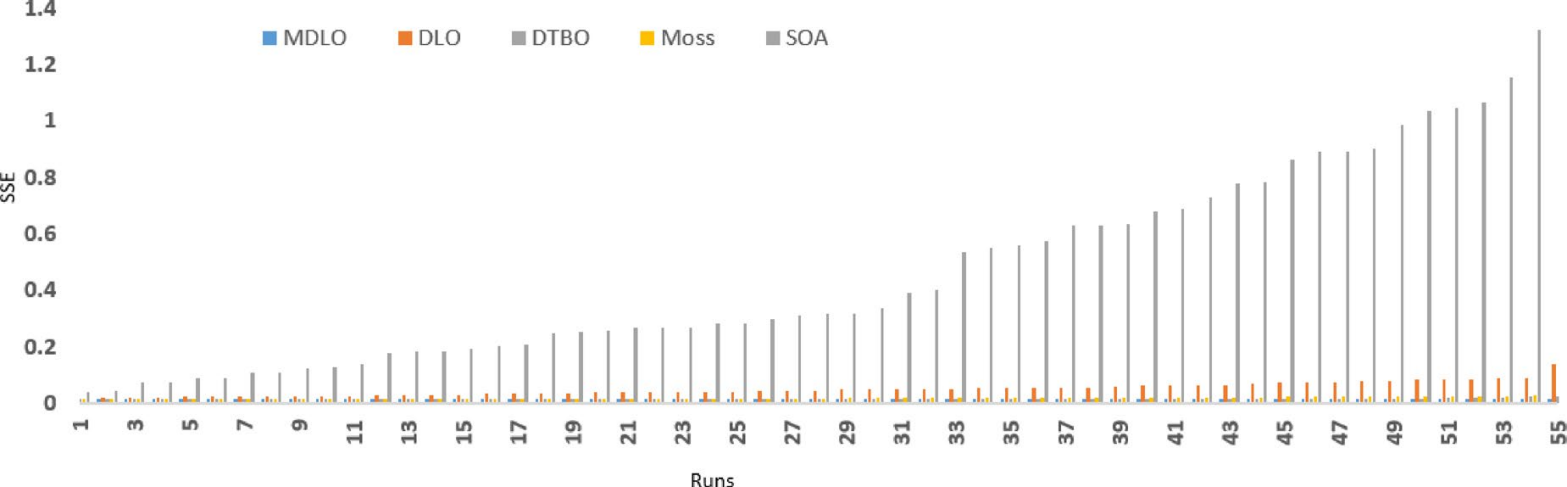
Separate Runs for the proposed MDLO and recently developed techniques for the BCS500W Stack.

The relative optimal SSE value for the MDLO is computed in 55 separate runs as illustrated in Fig. 4. It is evident that MDLO performed better than the newly created methods, which include the conventional DLO, SOA, Moss, and DTBO.

**Fig. 5:**
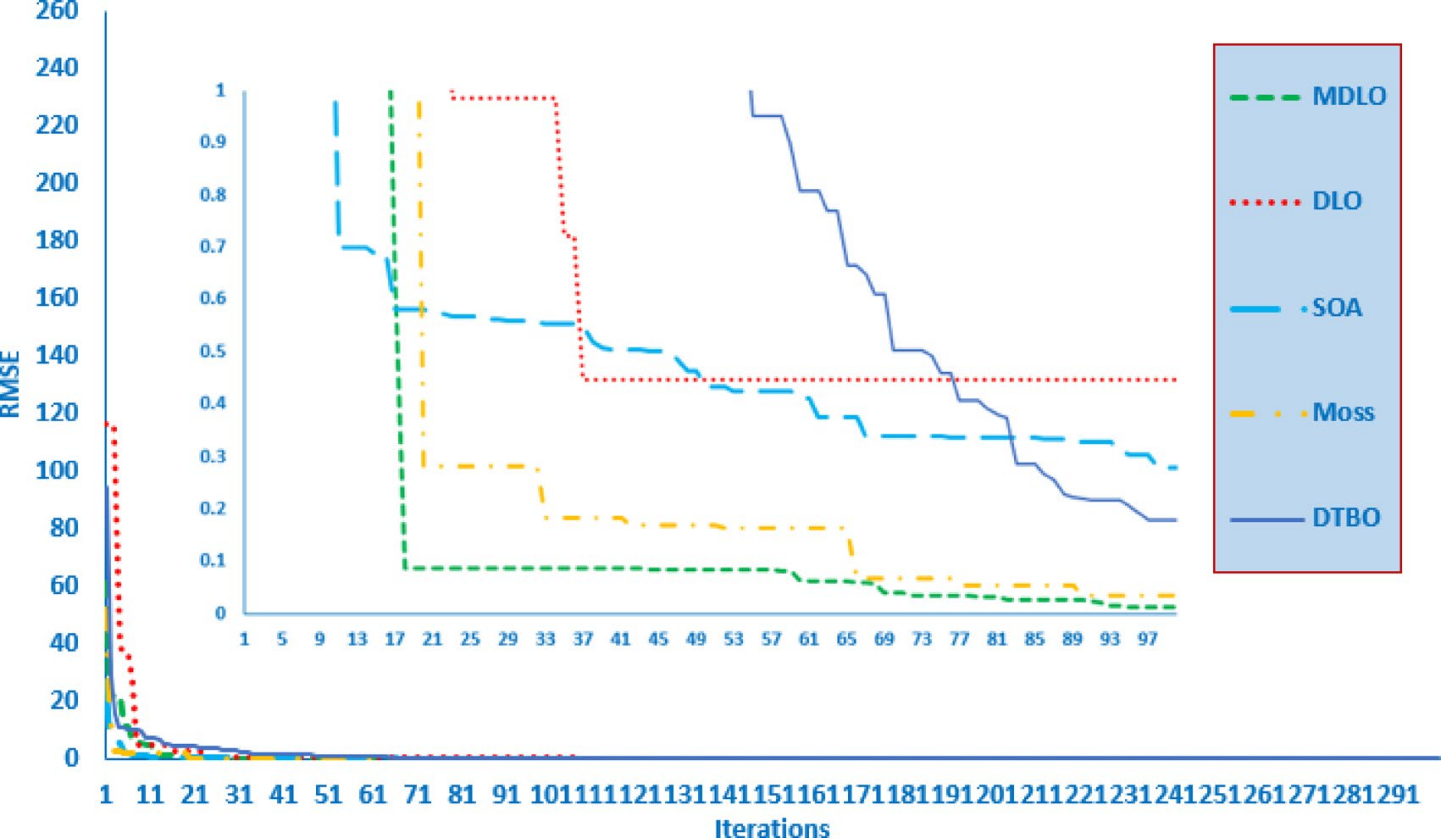
Convergence characteristics of the suggested MDLO versus recently developed techniques for BCS500W Stack.

Figure 5 demonstrates the convergence characteristics of the suggested MDLO. It is evident that MDLO outperforms the conventional DLO, SOA, Moss, and DTBO algorithms by quickly converging toward the best solution and achieving the minimum SEE value of 0.01169 in less than 69 iterations. This rapid convergence behavior demonstrates MDLO’s better exploitation capabilities and efficiency in obtaining high-quality solutions with little computational effort.

**Fig. 6:**
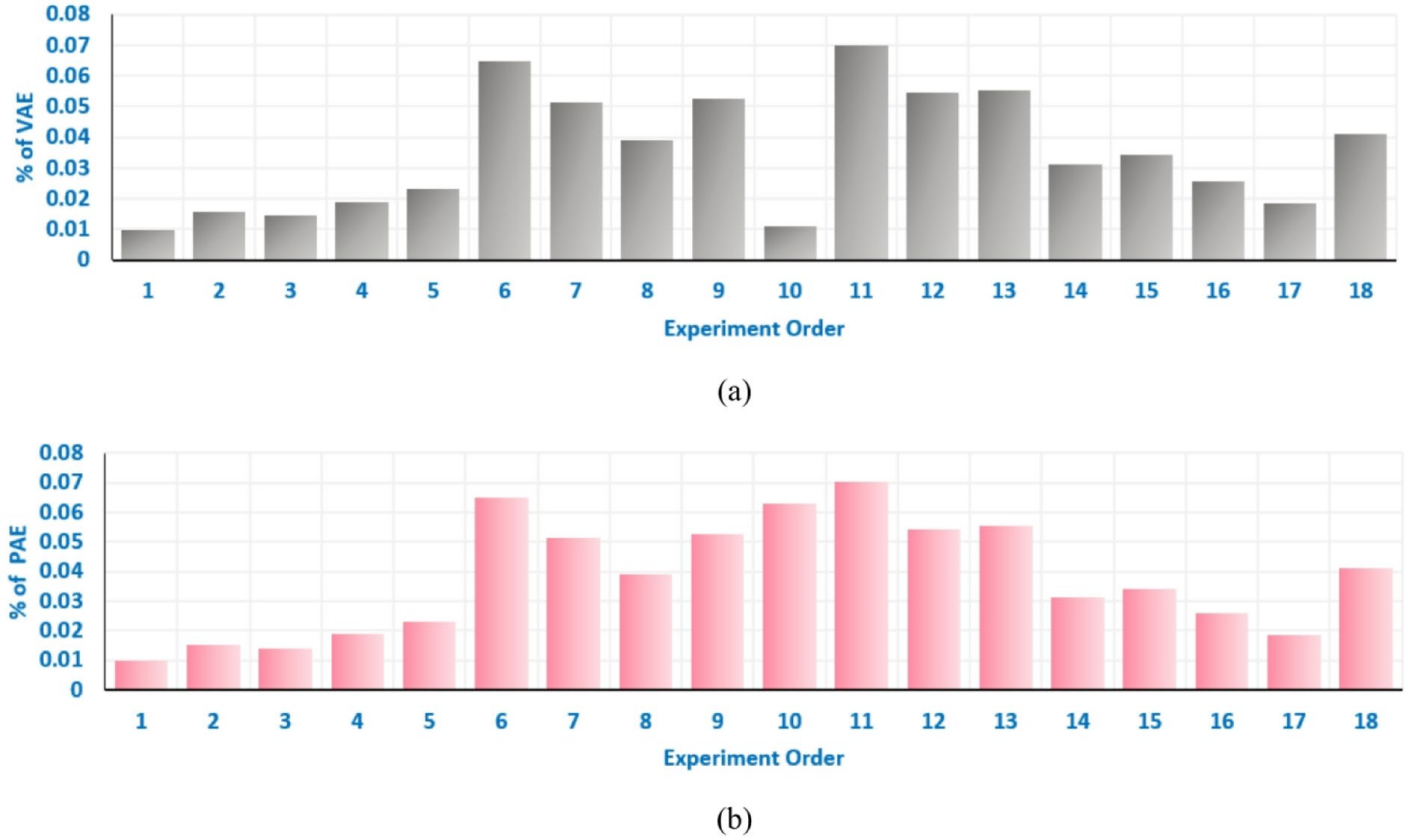
The percentage of the individual absolute errors between the simulated and experimental voltage and power of the BCS500W Stack.

Figure 6 (a, b) demonstrates a very close agreement between the simulated and experimental voltage and power of the BCS500W stack across the entire operating range.

The experimental and simulated voltage, current and power and the associated individual absolute errors between the simulated and experimental voltage and power are attached in Table A.1 in the Appendix A.

The percentage of the individual absolute errors for both voltage and power remain consistently low, with minimum values of about 0.0097% and maximum values not exceeding 0.0701%. Such small deviations indicate that the developed simulation model accurately captures the real stack behavior under different load conditions. The low VAE and PAE values confirm the high reliability and validity of the proposed model for predicting the electrical performance of the BCS500W stack.

**Fig. 7:**
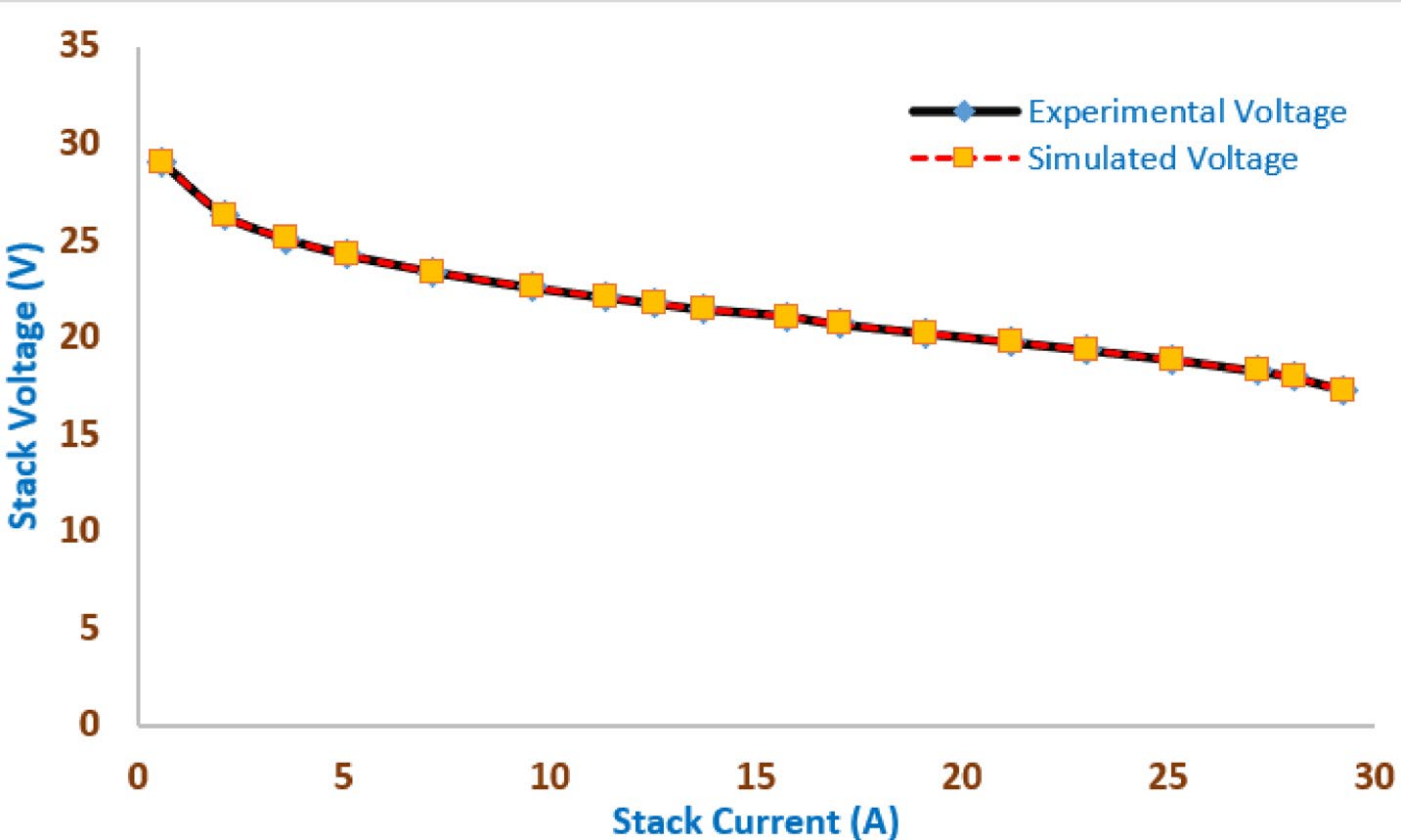
Experimental and simulated voltage points of BCS500W Stack obtained by MDLO versus stack current.

**Fig. 8:**
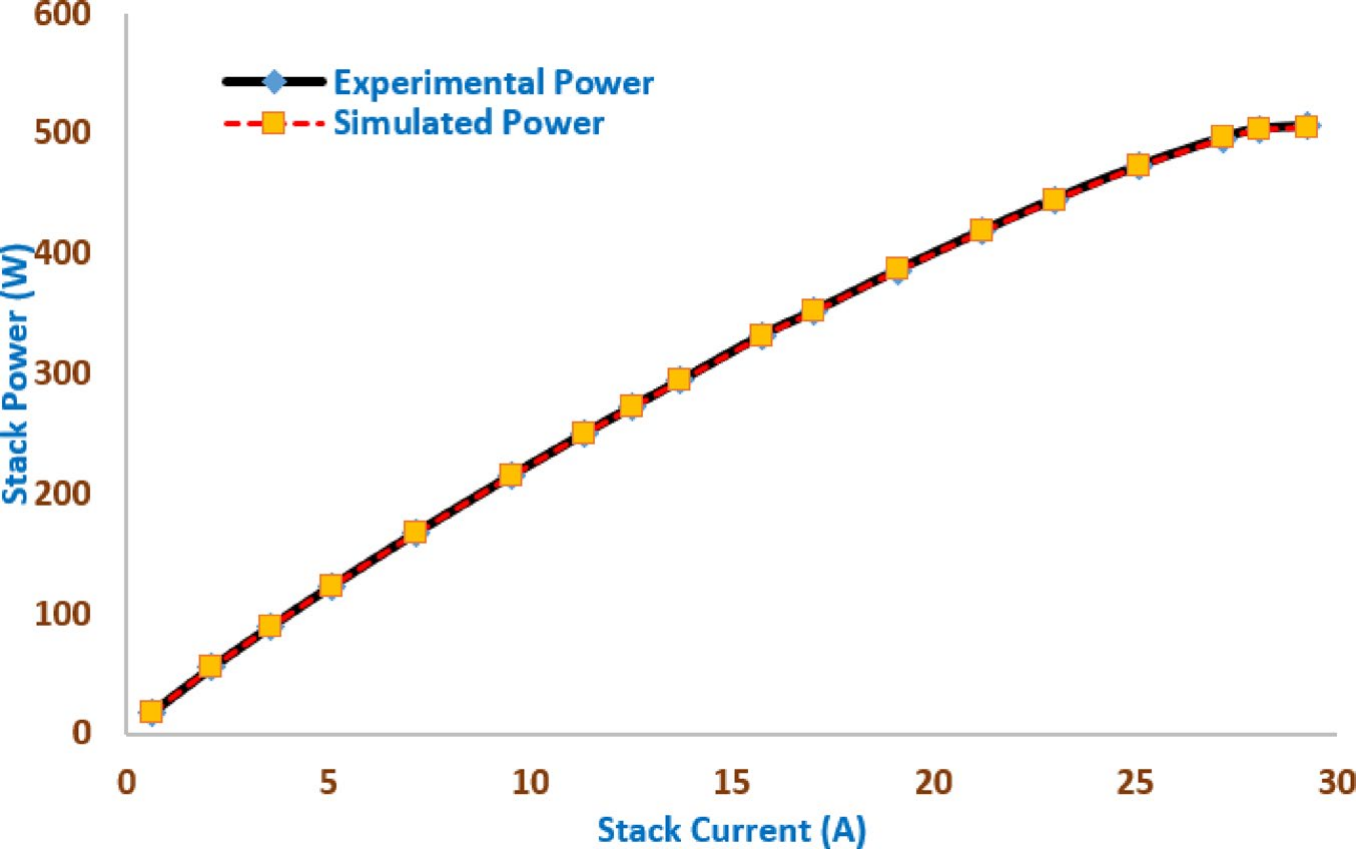
Experimental and simulated power points of BCS500W Stack obtained by MDLO versus stack current.

Figures 7 and 8 depict the experimental and simulated stack power and voltage points, respectively, in relation to the BCS500W Stack’s current as determined by MDLO. Figures 7 and 8 reveal remarkable matching between the measured and simulated V/I and P/I curves of 18 different points of the BCS500W Stack, respectively. The results highlight how accurately MDLO estimates power and current across a range of voltage values, since the modeling generated by MDLO matches precisely the experimental findings.

The suggested MDLO has been successful in determining the optimal values of the PEMFC’s unknown seven parameters after considering the set restrictions with a lower best SSE, according to the aforementioned demos for the analyzed cases. The exceptional performance of the MDLO and its outstanding design by its developers are reflected in the superiority of the MDLO-based PEMFC model over other optimization methods-based models. Additionally, as the simulation results show, it has a low number of parameters that need to be modified or fine-tuned together with a high and smooth convergence speed.

### 250 W PEMFC Stack

This stack consists of 24 series-connected cells with a membrane thickness of 178 μm, *M*_*A*_ = 27 *cm*^*2*^, a maximum current density of 680 *mA*/ *cm*^*2*^, and 23 *A* thermal current. The proposed MDLO is revealed to identify the ideal stack settings that produce the best PEMFC model values, as demonstrated in Table 4. As manifested in this table, the MDLO attained a small value of SSE of 0.331348, which is lower than the conventional DLO, SOA, Moss, and DTBO, which attain SSE values of 0.33411, 0.331364, 0.331415, and 0.333136, respectively. Furthermore, to exemplify the MDLO robustness, Table 4 characterizes the comparison between new reported techniques and the MDLO. The reported techniques are WOA^66^, grey wolf optimizer (GWO)^70^, Multi-verse optimizer (MVO)^71^, grasshopper optimizer (GHO)^72^, VSDE^62^, neural network optimizer (NNO)^73^. In terms of SSE, it is evident that the MDLO surpasses the conventional DLO and both the published technique and the new techniques in this study.

**Table 4:** The parameters extracted from the 250 W Stack using the proposed MDLO compared with the conventional DLO and reported techniques.

Technique	ξ_1_ (V)	ξ_2_$\:\times\:$10^−4^ (V/K)	ξ _3_$\:\times\:$10^−5^ (V/K)	ξ _4_$\:\times\:$10^−4^(V/K)	λ	*R*_c_(mΩ)	β(V)$\:\times\:$10^− 2^	SSE
MDLO	-1.056573666	28.8903	3.87809	-1.73822	14.42086586	0.100000	1.37908	0.331348
DLO	-1.074568089	37.0293	9.36296	-1.75035	16.12059187	0.200920	1.64644	0.33411
SOA	-0.926704241	26.1008	4.59378	-1.72114	14.94872586	0.310414	1.44860	0.331364
Moss	-1.171338954	32.2017	3.86053	-1.72670	15.61081754	0.488264	1.46067	0.331415
DTBO	-0.951874813	30.4293	7.15661	-1.74464	15.878678	0.426459	1.53358	0.333136
WOA[67]	-0.9565	32.221	8.2328	-1.75410	20.4470	0.1082	1.5200	0.3372
GWO[71]	-1.0564	37.953	9.8000	-1.17550	23.000	0.1088	1.3600	1.1505
MVO[72]	-0.9182	31.299	8.7031	-1.80253	15.1921	0.4223	1.800	3.5846
GHO [73]	-0.8532	31.237	9.8000	-1.70112	18.3441	0.1000	1.36	0.3395
VSDE[62]	01.1921	31.99	3.7990	18.7000	22.817	0.1202	2.903	1.0526
NNO[74]	−0.97997	36.946	9.0871	−1.62820	23.0000	0.1000	1.36	0.8536

Table 5 presents a statistical comparison of various methods to assess the effectiveness of the suggested MDLO. The effectiveness of the suggested MDLO in comparison to other revolutionary optimizers for calculating the parameters of the 250 W fuel cell stack is displayed in Table 5. The techniques are WOA^66^, GWO^70^, MVO^71^, GHO^72^, VSDE^62^. The statistical results presented in this table clearly demonstrate the superiority of the proposed MDLO for the 250 W stack. MDLO achieves the lowest best value of 0.331348 and the smallest mean value of 0.331348 among the conventional DLO and all compared optimizers, indicating high solution accuracy. Moreover, MDLO exhibits an extremely low standard deviation of 7.73$\:\times\:10$^-09^, which reflects excellent robustness and stable convergence behavior over repeated runs. In contrast, most reported optimizers show noticeably higher mean and worst-case values, accompanied by significantly larger standard deviations, revealing sensitivity to initial conditions and premature convergence. These results confirm that the proposed MDLO offers a more reliable and efficient optimization performance for the 250 W stack.

**Fig. 9:**
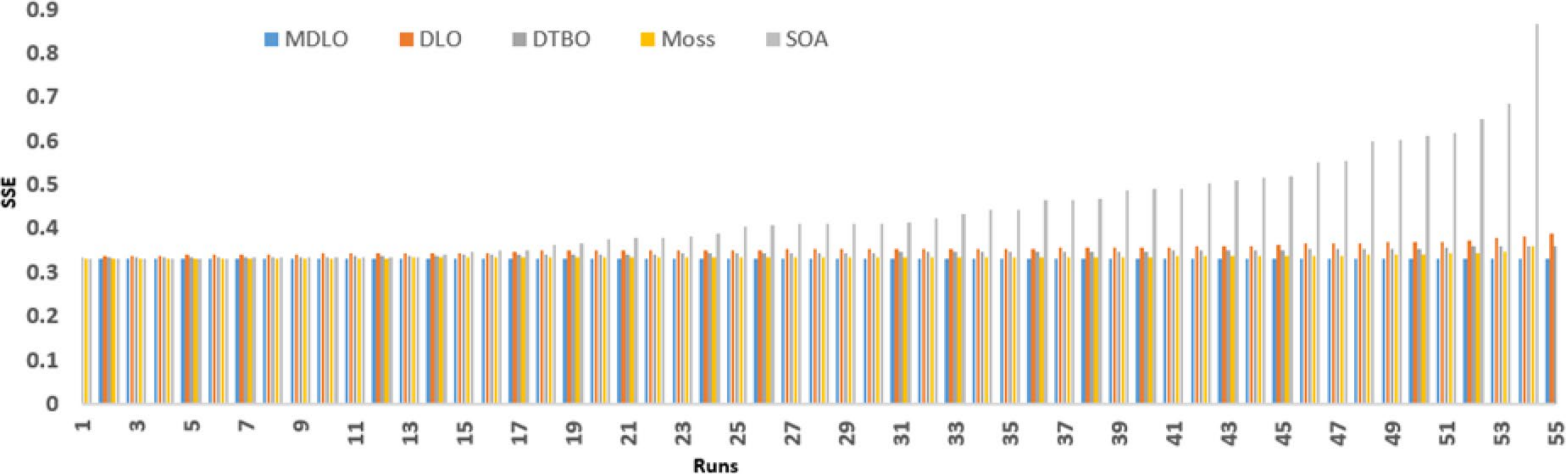
Separate Runs for the proposed MDLO and recently developed techniques for 250 W Stack.

The relative optimal SSE value for the MDLO is computed in 55 separate runs for 250 W Stack as illustrated in Fig. 9. It is evident that MDLO performed better than the newly created methods, which include the conventional DLO, SOA, Moss, and DTBO.

**Table 5:** The statistical comparison of the proposed MDLO with various methods for the 250 W.

Technique	Best	Mean	Worst	STD
MDLO	0.331348	0.331348	0.331348	7.73$\:\times\:10$^-09^
DLO	0.33411	0.353696	0.387404	1.1549$\:\times\:$*10*^*-2*^
DTBO	0.333136	0.34487	0.359429	7.127$\:\times\:$*10*^*-3*^
Moss	0.331415	0.336519	0.389066	8.629$\:\times\:$*10*^*-3*^
SOA	0.331364	0.446461	0.941215	1.30858$\:\times\:$*10*^*-1*^
WOA[67]	0.3372	-	0.5029	4.93$\:\times\:$*10*^*-2*^
GWO	1.1505	-	1.2329	9.21$\:\times\:$*10*^*-2*^
MVO	3.5846	-	11.420	200.9
GHO [73]	0.3395	-	0.4986	4.67$\:\times\:$*10*^*-2*^
VSDE	1.0526	-	1.1875	1.768$\:\times\:$*10*^*-1*^

**Fig. 10:**
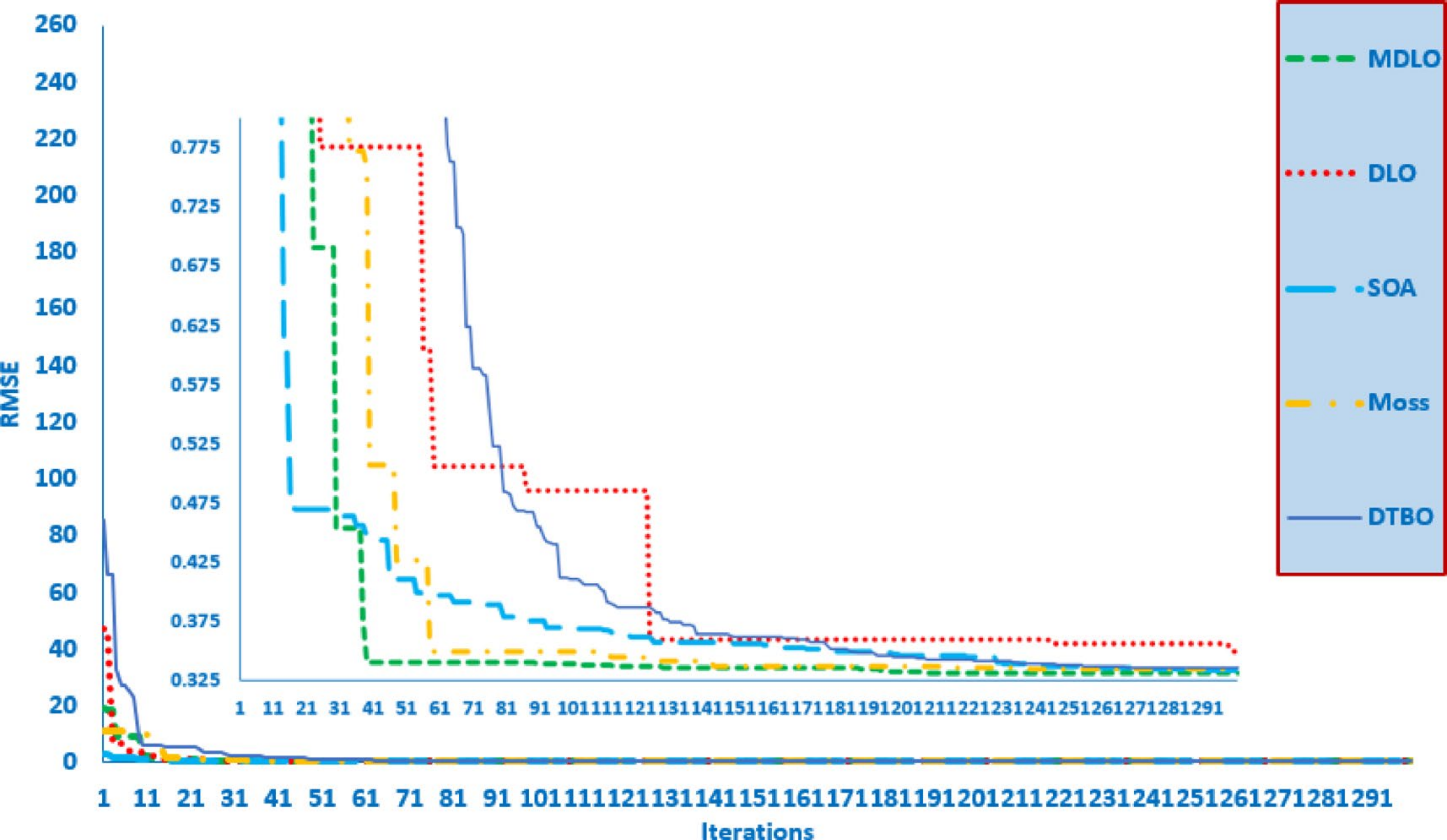
Convergence characteristics of the suggested MDLO versus recently developed techniques for the 250 W Stack.

Figure 10 demonstrates the convergence characteristics of the suggested MDLO. It is evident that MDLO outperforms the conventional DLO, SOA, Moss, and DTBO algorithms by quickly converging toward the best solution and achieving the minimum SEE value of 0.331348 in less than 38 iterations. This quick convergence behaviour indicates MDLO’s superior exploitation skills and efficiency in generating high-quality solutions with minimal computing effort.

**Fig. 11:**
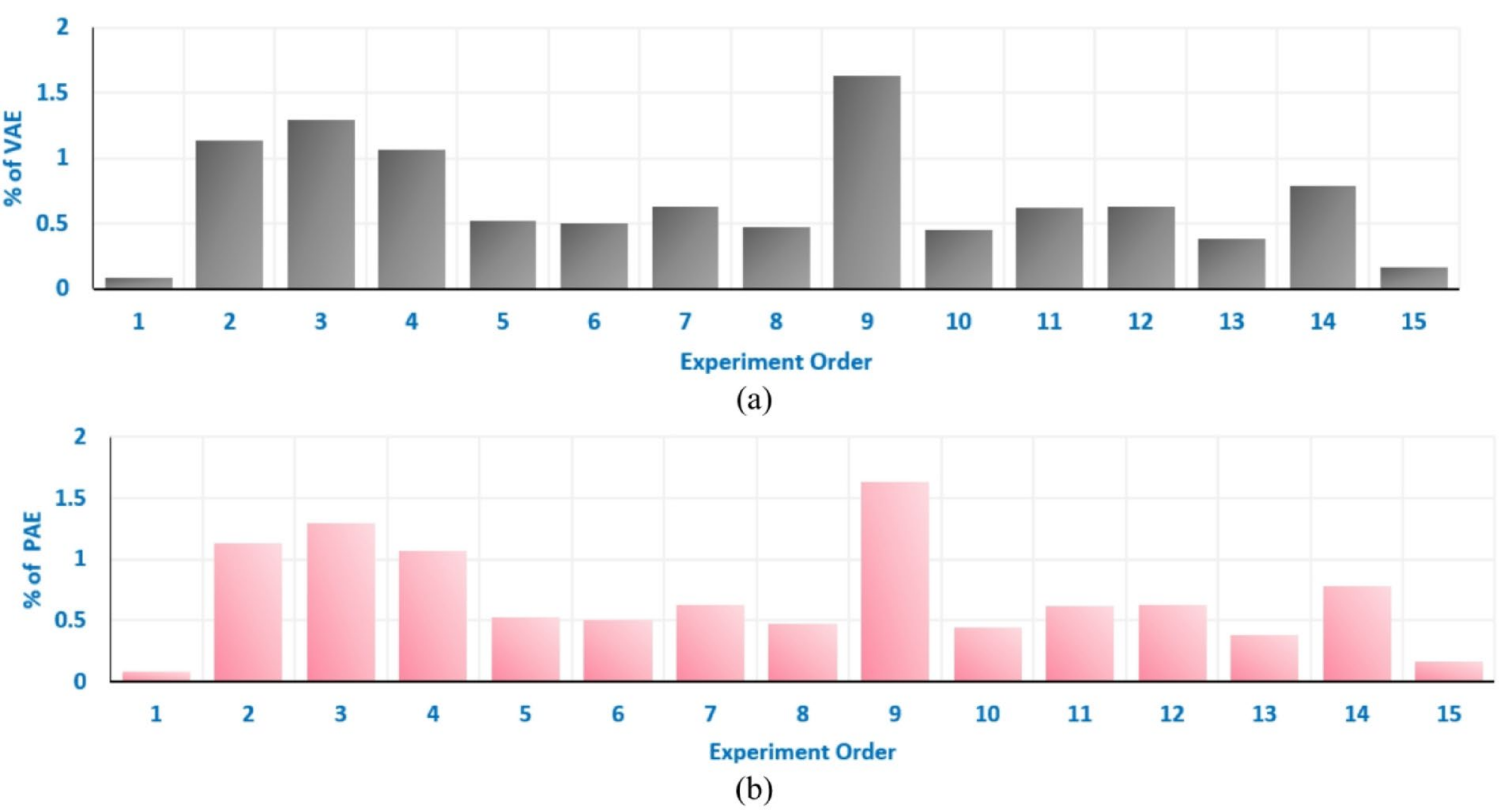
The percentage of the individual absolute errors between the simulated and experimental voltage and power of the 250 W Stack.

Figure 11 (a, b) demonstrates a very close agreement between the simulated and experimental voltage and power of the 250 W stack across the entire operating range. The experimental and simulated voltage, current and power and the associated individual absolute errors between the simulated and experimental voltage and power are attached in Table A.2 in the Appendix A. The percentage of the individual absolute errors for both voltage and power remain consistently low, with minimum values of about 0.088% and maximum values not exceeding 1.63%. Such small deviations indicate that the developed simulation model accurately captures the real stack behavior under different load conditions. The low VAE and PAE values confirm the high reliability and validity of the proposed model for predicting the electrical performance of the 250 W stack.

**Fig. 12:**
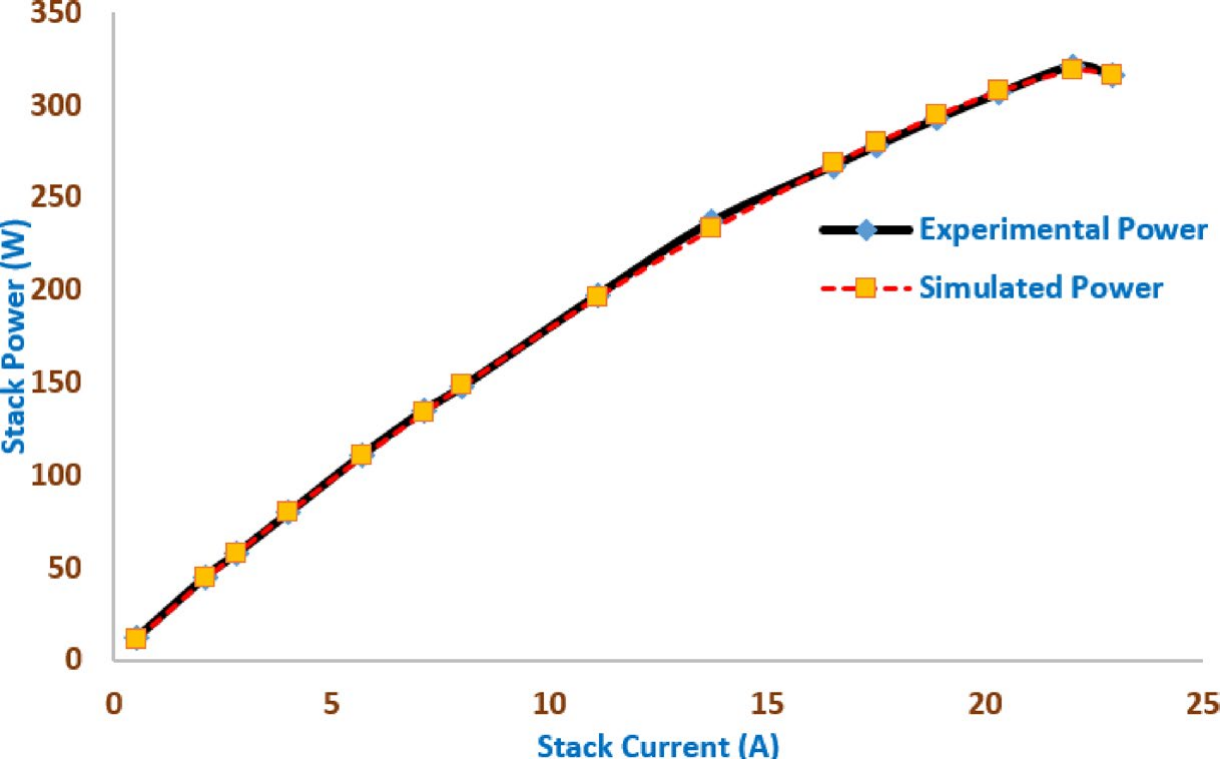
The experimental and simulated stack power points of the 250 W Stack obtained by MDLO versus stack current.

**Fig. 13:**
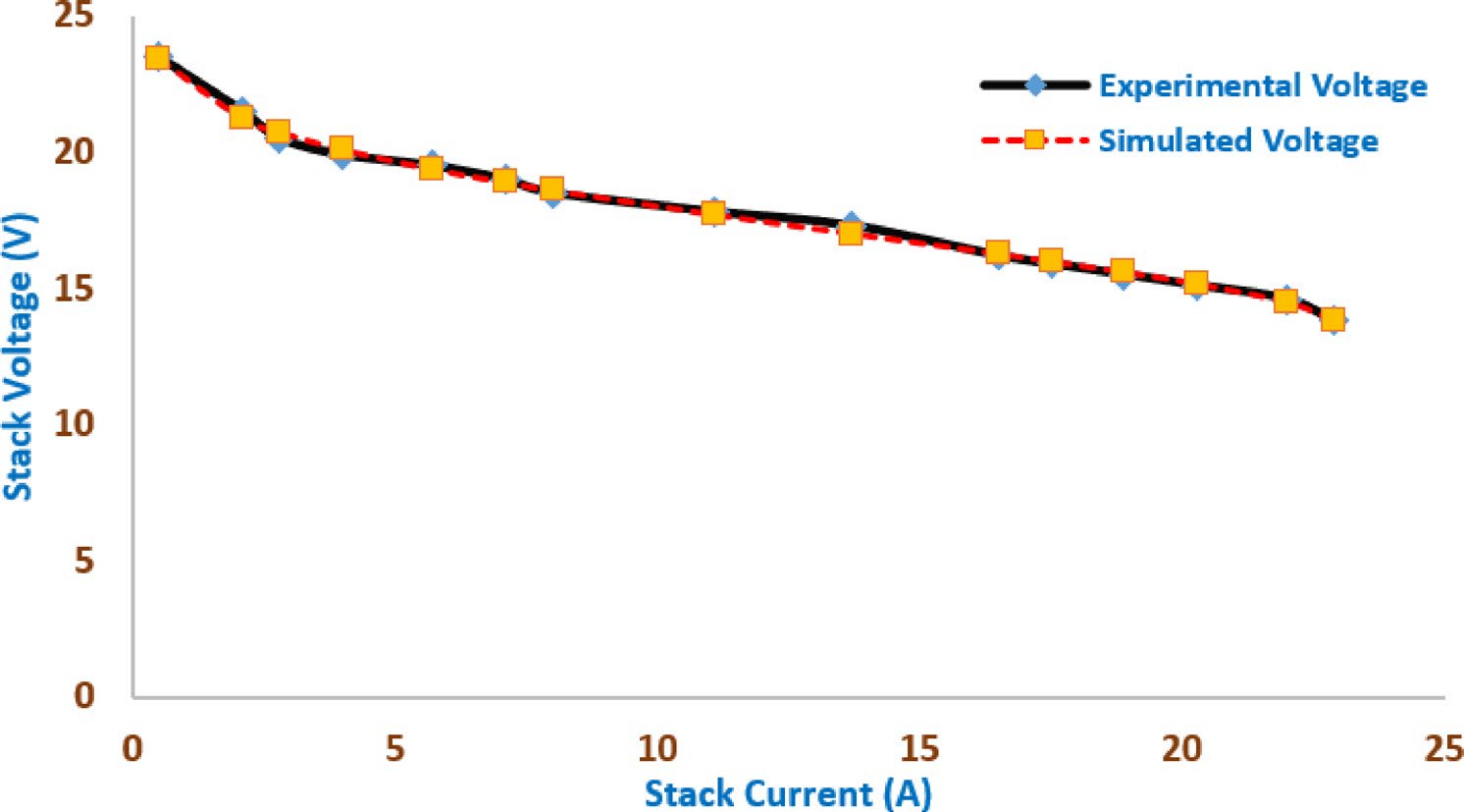
The experimental and simulated stack voltage points of the 250 W Stack obtained by MDLO versus stack current.

Figures 12 and 13 depict the experimental and simulated stack power and voltage points, respectively, in relation to the 250 W Stack’s current as determined by MDLO. Figures 12 and 13 reveal remarkable matching between the measured and simulated V/I and P/I curves of 15 different points of the 250 W Stack, respectively. The results highlight how accurately MDLO estimates power and current across a range of voltage values, since the modeling generated by MDLO matches precisely the experimental findings.

### NedStack PS6 PEMFC stacks

This stack of 6 kW rated power consists of 65 series-connected cells with a maximum current density of 1125 *mA*/ *cm* ^*2*^a membrane thickness of 178 μm, their areas (240 cm^2^)^2^, . The proposed MDLO is revealed to identify the ideal stack settings that produce the best PEMFC model values, as demonstrated in Table 6. As manifested in this table, the MDLO attained a small value of SSE of 2.100246, which is lower than the conventional DLO, SOA, Moss, and DTBO, which attain SSE values of 2.367099, 2.100246, 2.101393, and 2.330605, respectively. Furthermore, to exemplify the MDLO robustness, Table 6 characterizes the comparison between new reported techniques and the MDLO. The reported techniques are GOA^22^, ALO^22^, WOA^66^, MFO)^65^, , MVO^22^, SSA^67^, VSA^62^. In terms of SSE, it is evident that the MDLO surpasses the conventional DLO and both the published technique and the new techniques in this study.

Table 7 presents a statistical comparison of various methods to assess the effectiveness of the suggested MDLO. The effectiveness of the suggested MDLO in comparison to other revolutionary optimizers for calculating the parameters of the 250 W fuel cell stack is displayed in Table 7. The techniques are WOA^66^, GWO^70^, MVO^71^, GHO^72^, VSDE^62^. The statistical results presented in this table clearly demonstrate the superiority of the proposed MDLO for the 250 W stack. MDLO achieves the lowest best value of 2.100246and the smallest mean value of 2.100263 among the conventional DLO and all compared optimizers, indicating high solution accuracy. Moreover, MDLO exhibits an extremely low standard deviation of 1.0952$\:\times\:10$^-04^, which reflects excellent robustness and stable convergence behavior over repeated runs. In contrast, most reported optimizers show noticeably higher mean and worst-case values, accompanied by significantly larger standard deviations, revealing sensitivity to initial conditions and premature convergence. These results confirm that the proposed MDLO offers a more reliable and efficient optimization performance for the NedStack PS6 stack.

**Table 6:** The parameters extracted from the NedStack PS6 Stack using the proposed MDLO compared with the conventional DLO and reported techniques.

Technique-34rg0uj	ξ_1_ (V)	ξ_2_0.10^− 4^(V/K)	ξ_2_0.10^− 5^(V/K)	ξ_2_0.10^− 5^(V/K)	λ	*R*_c_(mΩ)	β(V) 10^− 2^	SSE
MDLO	-1.014701376	31.88749	6.01416	-9.5400	13.32302101	0.1000	1.36	2.100246
DLO	-1.125818265	33.2265	4.65558	-9.5400	13.32314778	0.1000	1.3600007	2.367099
SOA	-0.890579293	25.73758	4.20493	-9.5400	13.35072969	0.100012	1.4011774	2.100246
Moss	-0.985808477	32.62036	7.14239	-9.54375	14.09595162	0.115112	2.0932656	2.101393
DTBO	-1.017983338	34.25378	7.61049	-9.54001	21.57748163	0.271548	5.1092292	2.330605
VSA[62]	-0.8946	33.480	9.7500	-9.5400	13.0000	0.103	4.2900	2.34260
GOA[22]	-0.8546	32.687	9.8000	-9.5400	14.2349	0.124	1.8900	2.2185
WOA[67]	-0.8534	24.149	3.6200	-9.5400	22.9704	0.517	1.3800	4.0489
MFO[66]	-0.8532	31.364	8.8900	-9.5400	13.4656	0.100	1.3600	2.1459
ALO[22]	-0.9836	27.815	3.6200	-9.5400	13.9723	0.137	1.4100	2.2034
SSA[68]	-0.9894	3.3286	7.4100	-9.5400	20.5477	0.256	4.2600	2.5711
MVO[22]	-1.0394	3.2439	5.7700	-9.5400	16.1317	0.171	2.9000	2.3632

**Table 7:** The statistical comparison of the proposed MDLO with various methods for the NedStack PS6 Stack.

Technique	Best	Mean	Worst	STD
MDLO	2.100246	2.100263	2.101041	1.0952$\:\times\:10$^−04^
DLO	2.367099	2.886138	4.284251	3.0534$\:\times\:10$^−01^
SOA	2.100246	2.139328	2.381238	6.1467$\:\times\:10$^−02^
Moss	2.101393	2.257391	2.899141	1.4760$\:\times\:10$^−01^
DTBO	2.330605	2.514104	2.875531	1.2235$\:\times\:10$^−01^
MFO[66]	2.1459	3.0579	3.6555	6.947$\:\times\:10$^−01^
ALO[22]	2.2034	3.0480	4.1209	7.886$\:\times\:10$^−01^
WOA[67]	4.0489	56.5835	200.0259	84.5920
GOA[22]	2.2185	3.1793	4.0331	7.926$\:\times\:10$^−01^
SSA[68]	2.5711	9.3027	25.9350	9.8074
MVO[22]	2.3632	3.7385	4.3803	8.221$\:\times\:10$^−01^

**Fig. 14:**
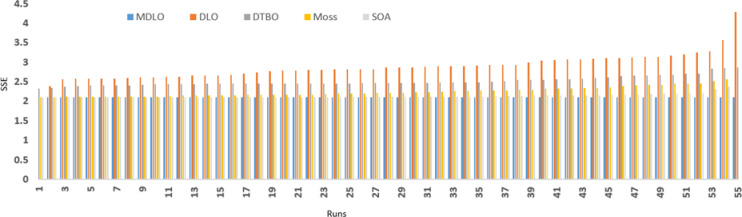
Separate Runs for the proposed MDLO and recently developed techniques for the NedStack PS6 Stack.

The relative optimal SSE value for the MDLO is computed in 55 separate runs for NedStack PS6 Stack as illustrated in Fig. 14. It is evident that MDLO performed better than the newly created methods, which include the conventional DLO, SOA, Moss, and DTBO.

**Fig. 15:**
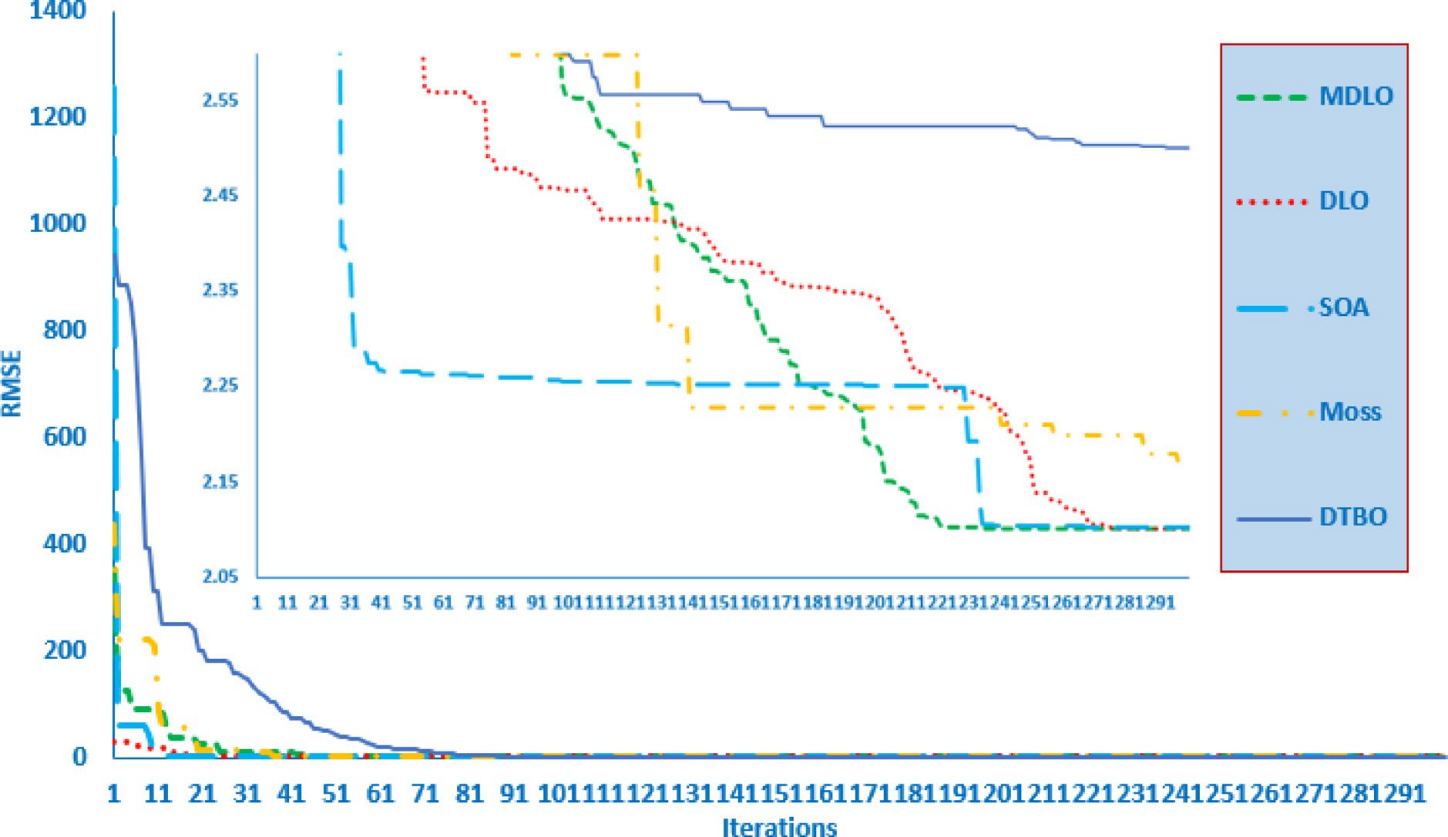
Convergence characteristics of the suggested MDLO versus recently developed techniques for NedStack PS6 Stack.

Figure 15 demonstrates the convergence characteristics of the suggested MDLO. It is evident that MDLO outperforms the conventional DLO, SOA, Moss, and DTBO algorithms by quickly converging toward the best solution and achieving the minimum SEE value of 2.100246 in less than 221 iterations. This rapid convergence behavior demonstrates MDLO’s better exploitation capabilities and efficiency in obtaining high-quality solutions with little computational effort.

**Fig. 16:**
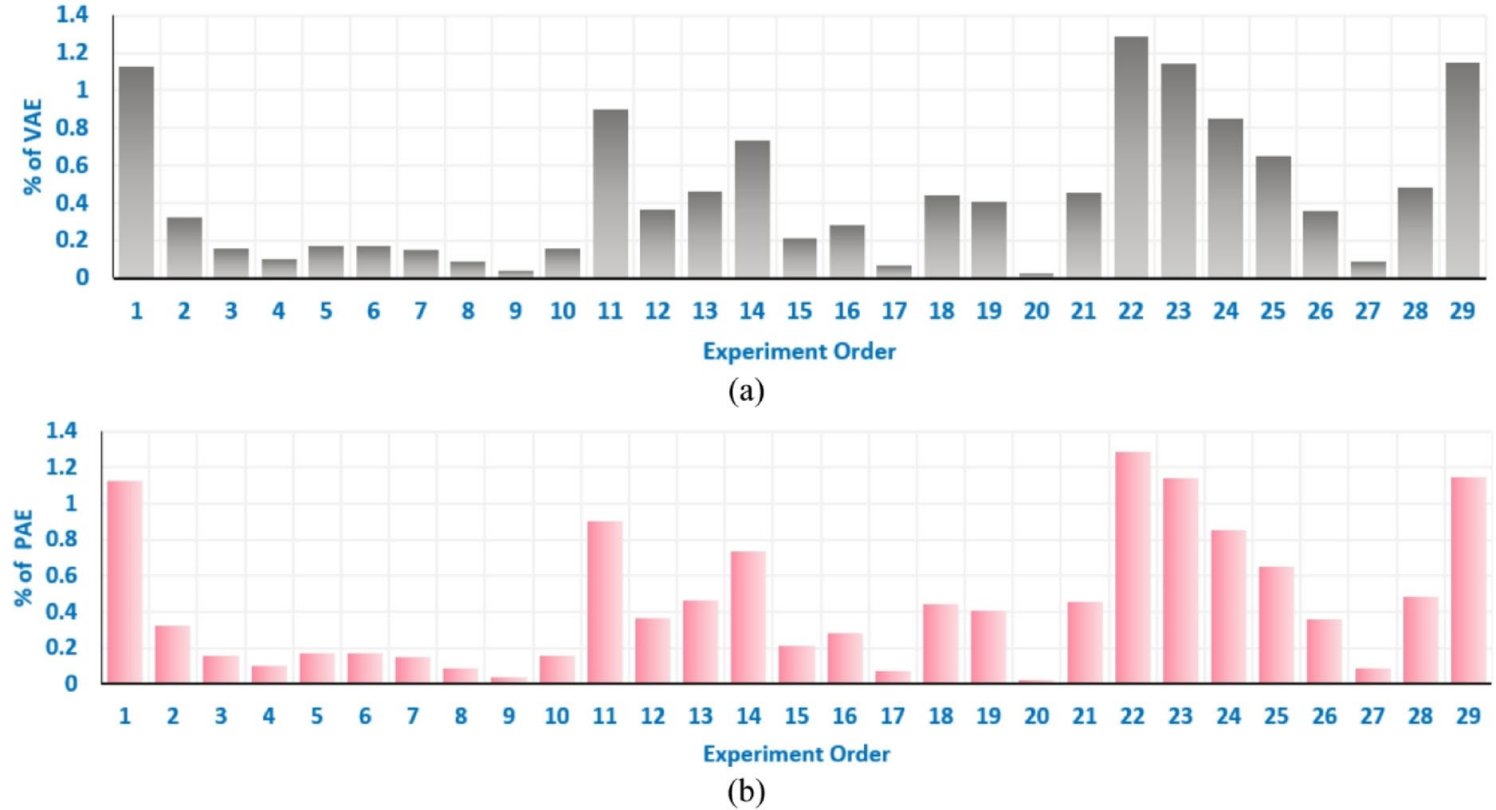
The percentage of the individual absolute errors between the simulated and experimental voltage and power of the NedStackPS6 Stack.

Figure 16 (a, b) demonstrates a very close agreement between the simulated and experimental voltage and power of the NedStack PS6 stack across the entire operating range. The experimental and simulated voltage, current and power and the associated individual absolute errors between the simulated and experimental voltage and power are attached in Table A.3 in the Appendix A. The percentage of the individual absolute errors for both voltage and power remains consistently low, with minimum values of about 0.025% and maximum values not exceeding 1.285%. Such small deviations indicate that the developed simulation model accurately captures the real stack behavior under different load conditions. The low VAE and PAE values confirm the high reliability and validity of the proposed model for predicting the electrical performance of the NedStack PS6 stack.

**Fig. 17:**
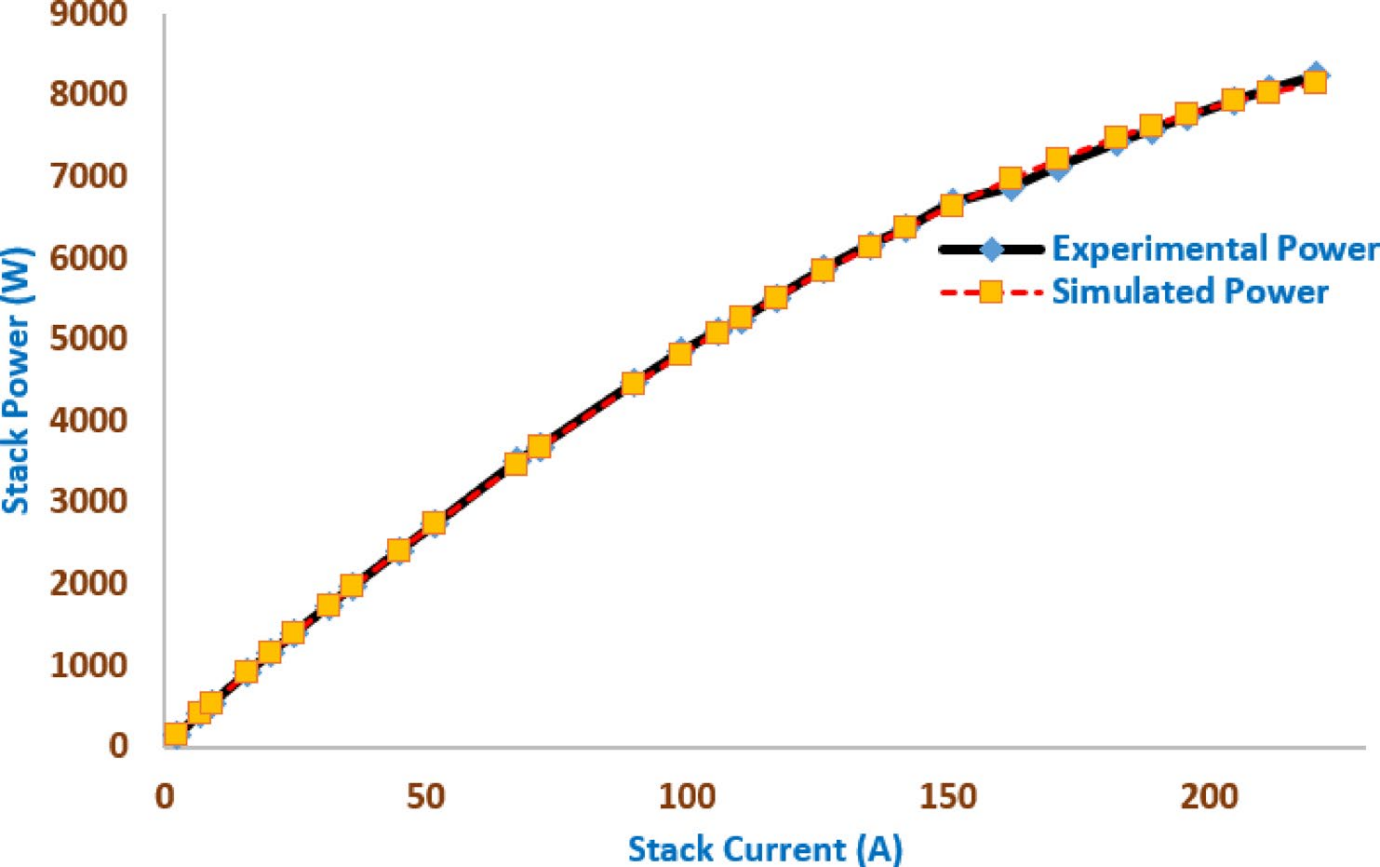
The experimental and simulated stack power points of the NedStackPS6 Stack obtained by MDLO versus stack current.

**Fig. 18:**
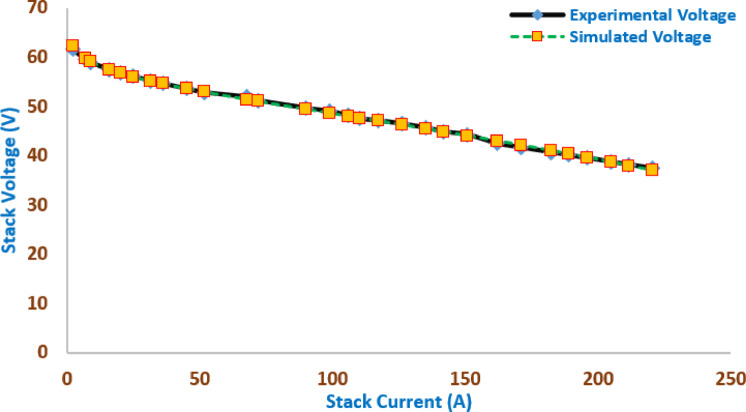
The experimental and simulated stack voltage points of the NedStackPS6 Stack obtained by MDLO versus stack current.

Figures 17 and 18 depict the experimental and simulated stack power and voltage points, respectively, in relation to the NedStackPS6 Stack’s current as determined by MDLO. Figures 17 and 18 reveal remarkable matching between the measured and simulated V/I and P/I curves of 29 different points of the NedStackPS6 Stack, respectively. The results highlight how accurately MDLO estimates power and current across a range of voltage values, since the modeling generated by MDLO matches precisely the experimental findings.

### Comprehensive statistical validation and robustness analysis

To statistically validate the comparative results, a Wilcoxon signed-rank test was performed over 55 independent runs. As reported in Table 8, all pairwise comparisons involving MDLO produced extremely small p-values (≈ 1.108 × 10⁻¹⁰), which are significantly lower than the standard significance threshold (α = 0.05), confirming statistically significant superiority over DLO, DTBO, Moss, and SOA across the 500 W, 250 W, and 6 kW PEMFC stacks. In multiple cases, the signed-rank statistic equals 0 (e.g., MDLO vs. DTBO, Moss, and SOA), indicating that MDLO outperformed the compared algorithm in nearly every run. Comparisons among the remaining algorithms also yielded p-values < 0.05, but the strongest and most consistent dominance is clearly in favor of MDLO.

**Table 8:** Wilcoxon Signed-Rank Test Results: Pairwise Comparison.

PEMFC stack	BCS 500		250 W		NedStack PS6	
Comparison	p-value	Signed-Rank Statistic	p-value	Signed-Rank Statistic	p-value	Signed-Rank Statistic
DLO vs. MDLO	1.108$\:\times\:10$^−10^	1540	1.108$\:\times\:10$^−10^	1540	1.108$\:\times\:10$^−10^	1540
DLO vs. DTBO	1.108$\:\times\:10$^−10^	1540	1.161$\:\times\:10$^−04^	1230	5.349$\:\times\:10$^−10^	1511
DLO vs. Moss	2.027$\:\times\:10$^−10^	1529	4.492$\:\times\:10$^−09^	1470	1.171$\:\times\:10$^−10^	1539
DLO vs. SOA	2.140$\:\times\:10$^−10^	12	2.113$\:\times\:10$^−06^	204	1.108$\:\times\:10$^−10^	1540
MDLO vs. DTBO	1.108$\:\times\:10$^−10^	0	1.108$\:\times\:10$^−10^	0	1.108$\:\times\:10$^−10^	0
MDLO vs. Moss	1.108$\:\times\:10$^−10^	0	1.108$\:\times\:10$^−10^	0	1.108$\:\times\:10$^−10^	0
MDLO vs. SOA	1.108$\:\times\:10$^−10^	0	1.108$\:\times\:10$^−10^	0	1.108$\:\times\:10$^−10^	0
DTBO vs. Moss	2.203$\:\times\:10$^−06^	205	4.264$\:\times\:10$^−08^	1424	1.790$\:\times\:10$^−09^	1488
DTBO vs. SOA	1.108$\:\times\:10$^−10^	0	7.489$\:\times\:10$^−08^	128	1.108$\:\times\:10$^−10^	1540
Moss vs. SOA	1.108$\:\times\:10$^−10^	0	7.056$\:\times\:10$^−09^	79	7.148$\:\times\:10$^−08^	1413

To further validate the robustness of the comparative analysis, the Friedman non-parametric test was conducted across the five algorithms in Table 9, indicating that the Friedman test further confirms these findings. The obtained p-values (9.074 × 10⁻⁴⁴ for BCS 500 W, 6.282 × 10⁻³³ for 250 W, and 7.541 × 10⁻⁴³ for 6 kW) overwhelmingly reject the null hypothesis of equivalent performance. MDLO consistently achieves a mean rank of 1.00 across all stacks, while the ranks of competing algorithms vary depending on stack scale. This demonstrates that MDLO maintains first-place performance regardless of system rating or landscape complexity.

**Table 9:** Friedman Test Results.

PEMFC Stack	Friedman *p*-value	DLO	Proposed MDLO	DTBO	Moss	SOA
BCS 500 W	9.074$\:\times\:10$^−44^	4.04	1.00	2.22	2.82	4.93
250 W	6.282$\:\times\:10$^−33^	3.93	1.00	3.40	2.36	4.31
NedStack PS6 (6 kW)	7.541$\:\times\:10$^−43^	4.91	1.00	3.95	2.98	2.16

Moreover, robustness was evaluated using variance and coefficient of variation (CV). As shown in Table 10, MDLO exhibits dramatically lower variance values across all stacks (5.41 × 10⁻¹⁰, 5.83 × 10⁻¹⁷, and 1.20 × 10⁻⁸) compared to DLO (5.59 × 10⁻⁴, 1.33 × 10⁻⁴, 9.32 × 10⁻²) and SOA (1.35 × 10⁻¹, 1.71 × 10⁻², 3.78 × 10⁻³). The corresponding CV values (0.0020, 0.0000, and 0.0001) confirm negligible performance fluctuation, while competing methods show significantly higher variability (e.g., SOA CV = 0.7732 for 500 W). These results indicate exceptional convergence stability of MDLO.

**Table 10:** Variance and Robustness Analysis.

PEMFC stack	BCS 500		250 W		NedStack PS6	
Algorithm	Variance	Coefficient of Variation (CV)	Variance	CV	Variance	CV
DLO	5.585540$\:\times\:10$^−04^	0.4827	1.333732$\:\times\:10$^−04^	0.0327	9.323285$\:\times\:10$^−02^	0.1058
Proposed MDLO	5.411398$\:\times\:10$^−10^	0.0020	5.832862$\:\times\:10$^−17^	0.0000	1.199367$\:\times\:10$^−08^	0.0001
DTBO	6.341314$\:\times\:10$^−06^	0.1862	5.079116$\:\times\:10$^−05^	0.0207	1.496853$\:\times\:10$^−02^	0.0487
Moss	2.278631$\:\times\:10$^−05^	0.2707	7.446565$\:\times\:10$^−05^	0.0256	2.178703$\:\times\:10$^−02^	0.0654
SOA	1.349314$\:\times\:10$^−01^	0.7732	1.712373$\:\times\:10$^−02^	0.2931	3.778140$\:\times\:10$^−03^	0.0287

To further quantify the reliability of the reported mean SSE values, non-parametric bootstrap confidence intervals (CIs) (95%) were computed for each algorithm and PEMFC stack as shown in Table 11. The tabulated CIs (95%) further reinforce reliability. As shown in Table 11, the proposed MDLO consistently exhibits the narrowest confidence intervals across all stacks, indicating extremely low dispersion and highly stable convergence behavior. For the BCS 500 W stack, MDLO’s CI [1.170063$\:\times\:10$^-02^, 1.171259$\:\times\:10$^-02^] is clearly separated from DTBO [1.292182$\:\times\:10$^-02^, 1.423366$\:\times\:10$^-02^] and Moss [1.643600$\:\times\:10$^-02^, 1.891439$\:\times\:10$^-02^], with no overlap. Similar tight intervals are observed for the 250 W ([3.313476$\:\times\:10$^-01^, 3.313476$\:\times\:10$^-01^]) and 6 kW ([2.100246, 2.100296]) stacks, confirming minimal estimation uncertainty.

**Table 11:** 95% Bootstrap Confidence Intervals for Mean Objective (SSE).

PEMFC stack	BCS 500	250 W	NedStack PS6
Algorithm	95% Bootstrap CI	95% Bootstrap CI	95% Bootstrap CI
DLO	[4.294297$\:\times\:10$^−02^, 5.540514$\:\times\:10$^−02^]	[3.507678$\:\times\:10$^−01^, 3.568332$\:\times\:10$^−01^]	[2.812068, 2.970989]
Proposed MDLO	[1.170063$\:\times\:10$^−02^, 1.171259$\:\times\:10$^−02^]	[3.313476$\:\times\:10$^−01^, 3.313476$\:\times\:10$^−01^]	[2.100246, 2.100296]
DTBO	[1.292182$\:\times\:10$^−02^, 1.423366$\:\times\:10$^−02^]	[3.429772$\:\times\:10$^−01^, 3.467274$\:\times\:10$^−01^]	[2.483457, 2.547281]
Moss	[1.643600$\:\times\:10$^−02^, 1.891439$\:\times\:10$^−02^]	[3.346633$\:\times\:10$^−01^, 3.390532$\:\times\:10$^−01^]	[2.220778, 2.298159]
SOA	[3.822848$\:\times\:10$^−01^, 5.766079$\:\times\:10$^−01^]	[4.142831$\:\times\:10$^−01^, 4.823372$\:\times\:10$^−01^]	[2.124657, 2.156839]

**Fig. 19:**
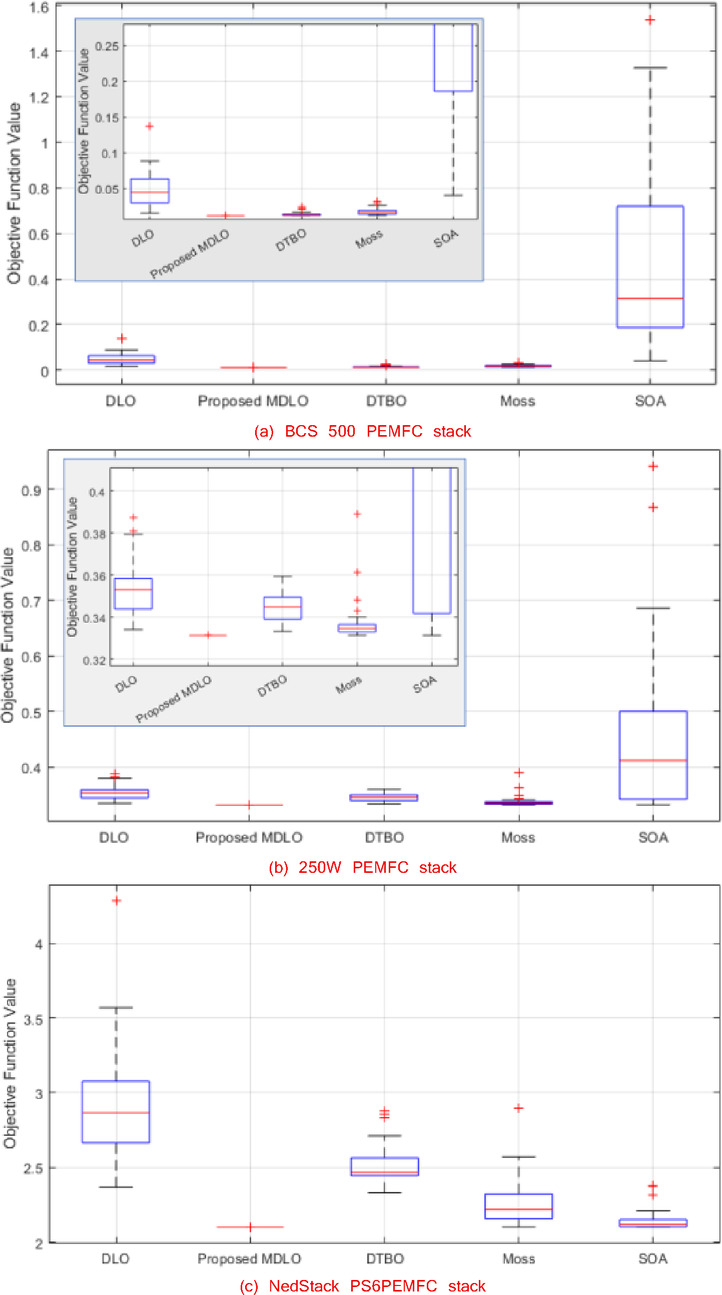
Boxplots of the compared algorithms for the three studied PEMFC stacks.

Otherwise, Fig. 19 visually supports these findings. MDLO consistently exhibits the lowest median SSE, the narrowest interquartile range, and minimal outliers across all three stacks. In contrast, DLO and SOA show wide dispersion and pronounced variability. The compact box structure of MDLO, particularly for the 250 W case, indicates highly consistent convergence across runs.

Based on the above analysis, the Wilcoxon test, Friedman ranking, variance/CV analysis, bootstrap confidence intervals, and boxplot distributions collectively confirm that MDLO’s superiority is statistically significant, highly stable, and reproducible across different PEMFC scales. The improvements are therefore not attributable to stochastic variation but reflect a robust enhancement in search dynamics.

### Computational complexity, runtime performance, and memory utilization analysis

Table 12 presents the mean computational time and memory consumption over 55 independent runs for the three investigated PEMFC stacks. It can be observed that DLO and Moss exhibit the lowest runtime (≈ 7 s), followed closely by the proposed MDLO (≈ 7–8 s), whereas SOA and DTBO require significantly longer execution times. In particular, DTBO shows the highest computational burden, with an average runtime approximately 2.4 times higher than MDLO. Although MDLO introduces a slight runtime increase compared to the original DLO due to its enhanced search dynamics, it remains within the same computational order and substantially faster than DTBO and SOA. Moreover, the elapsed time remains nearly constant across the 250 W, 500 W, and 6 kW stacks, confirming strong scalability with respect to system rating and dataset size.

**Table 12:** Computational time and memory consumption for the PEMFC stacks.

Algorithm	Mean computational time (s)			Memory consumption (MB)		
	BCS 500	250 W	NedStack	BCS 500	250 W	NedStack
DLO	7.04	6.77	7.57	3856	3856	3855
Proposed MDLO	7.98	7.28	8.11	3853	3853	3854
DTBO	18.96	18.37	19.94	3905	3905	3857
Moss	7.34	6.99	7.41	3910	3913	3913
SOA	11.67	11.77	11.94	3919	3920	3920

Regarding memory usage, all algorithms demonstrate comparable consumption (≈ 3.85–3.92 GB), indicating that the storage requirement is primarily governed by MATLAB runtime overhead and population storage rather than algorithmic structure. Importantly, the proposed MDLO does not introduce additional memory-intensive operators, maintaining similar memory complexity $\:O(population\:size\times\:Dimension)$to the other population-based methods. This confirms that the improved robustness and convergence stability achieved by MDLO are obtained without increasing memory burden, resulting in a favorable trade-off between computational efficiency, scalability, and optimization performance.

### Benchmark validation and robustness analysis using the speed reducer design problem

To further validate the effectiveness, robustness, and competitiveness of the proposed Modified DLO (MDLO), an additional comparative study was conducted using the classical Speed Reducer Design benchmark problem. This problem is widely recognized in engineering optimization due to its nonlinear objective function, multiple design constraints, and mixed variable interactions, making it suitable for assessing both convergence capability and solution stability.

The proposed MDLO was compared with several recent and established metaheuristic algorithms reported in the literature^74^, including: Water Uptake and Transport in Plants algorithm, 2025, (WUTP)^75^, Rime Optimization Algorithm, 2023, (RIME)^76–78^, Walrus Optimizer, 2023, (WO)^79^, PSO^80^, and Kangaroo Escape Algorithm, 2025, (KEA)^81^. The performance of these algorithms was evaluated on the Speed Reducer Design problem, a widely recognized benchmark in industrial design optimization^82^. The mathematical formulation of the speed reducer design problem is provided in Appendix B. All algorithms were executed for 50 independent runs under identical parameter settings to ensure statistical fairness and reliability.

Table 13 presents the optimal design variables and corresponding minimum fabrication cost obtained by each algorithm. It can be observed that the proposed MDLO achieves the lowest objective value (2994.424), matching or slightly outperforming the best results reported by other competitive methods such as PSO, WUTP, and KEA. Moreover, MDLO produces highly precise design variable values with minimal deviation from theoretical optima.

**Table 13:** Optimal design variables and best objective function values obtained by the compared algorithms for the speed reducer design problem.

Control Parameters	RIME	PSO	WUTP	WO	KEA	DLO	MDLO
*x*_*1*_	3.5	3.5	3.5	3.5001	3.5	3.500262	3.5
*x*_*2*_	0.7	0.7	0.7	0.7	0.7	0.700004	0.7
*x*_*3*_	17	17	17	17	17	17.00003	17
*x*_*4*_	7.3	7.3	7.3	7.3	7.3	7.32211	7.3
*x*_*5*_	7.7332	7.7153	7.7153	7.7249	7.7153	7.715866	7.71532
*x*_*6*_	3.3506	3.3505	3.3505	3.3506	3.3505	3.350926	3.350541
*x*_*7*_	5.2867	5.2867	5.2867	5.2867	5.2867	5.286712	5.286654
*F(x)*	2994.8939	2994.4245	2994.4254	2994.6967	2994.4245	2994.894	2994.424

Table 14 summarizes the statistical indicators over 50 runs, providing deeper insight into convergence stability and robustness. The proposed MDLO achieves the lowest minimum fitness value. It also acquires a mean fitness nearly identical to its minimum and the smallest maximum fitness. It obtains an exceptionally low standard deviation (0.000165). The extremely small standard deviation confirms that MDLO consistently converges to the global optimum with negligible dispersion. In contrast, PSO exhibits very high variability (standard deviation = 62.73), indicating instability and sensitivity to initialization. RIME and WUTP show moderate stability, whereas DLO demonstrates improved consistency compared to most algorithms but still does not match the robustness of MDLO. These findings clearly confirm the superior reliability and repeatability of the proposed method.

**Table 14:** Statistical performance metrics (minimum, mean, maximum, and standard deviation) for the speed reducer design problem.

Statistical indices	RIME	PSO	WO	WUTP	KEA	DLO	Proposed MDLO
Minimum Fitness	2994.894	2994.425	2994.697	2994.425	2994.425	2994.894	2994.424
Mean Fitness	3000.489	3073.803	2998.55	2997.196	2994.769	2995.446	2994.425
Maximum Fitness	3012.789	3197.598	3007.862	3032.007	2999.523	2996.606	2994.425
Standard deviation	4.6605	62.7313	3.4732	9.2744	0.9185	0.348085	0.000165

Table 15 presents bootstrap confidence intervals at 95%, 90%, 85%, and 80% confidence levels. The proposed MDLO exhibits an extremely narrow interval across all confidence levels, with virtually identical lower and upper bounds. This indicates highly concentrated convergence behavior and confirms the statistical reliability of the obtained solution.

Conversely, PSO demonstrates the widest confidence intervals, reflecting substantial variability and lower robustness. RIME and WUTP show moderate dispersion, while DLO presents improved stability but still with noticeably wider intervals compared to MDLO. The progressive narrowing of intervals from 95% to 80% further illustrates the consistency pattern, where MDLO remains practically unchanged, highlighting its deterministic-like convergence behavior.

**Table 15:** Bootstrap confidence intervals of the objective function values at different confidence levels for the speed reducer design problem.

CI	RIME	PSO	WO	WUTP	KEA	DLO	Proposed MDLO
95%	[2999.16-3001.81]	[3055.97-3091.63]	[2997.56-2999.54]	[2994.58-2999.83]	[2994.39-2995.15]	[2994.916-2996.068]	[2994.424-2994.425]
90%	[2999.38-3001.59]	[3058.93-3088.68]	[2997.73-2999.37]	[2995.00-2999.39]	[2994.45-2995.09]	[2995.006-2995.88]	[2994.424-2994.424]
85%	[2999.53-3001.45]	[3060.83-3086.78]	[2997.83-2999.27]	[2995.28-2999.11]	[2994.49-2995.05]	[2995.081-2995.81]	[2994.424-2994.424]
80%	[2999.63-3001.35]	[3062.28-3085.33]	[2997.91-2999.19]	[2995.49-2998.90]	[2994.52-2995.02]	[2995.174-2995.65]	[2994.424-2994.424]

**Fig. 20:**
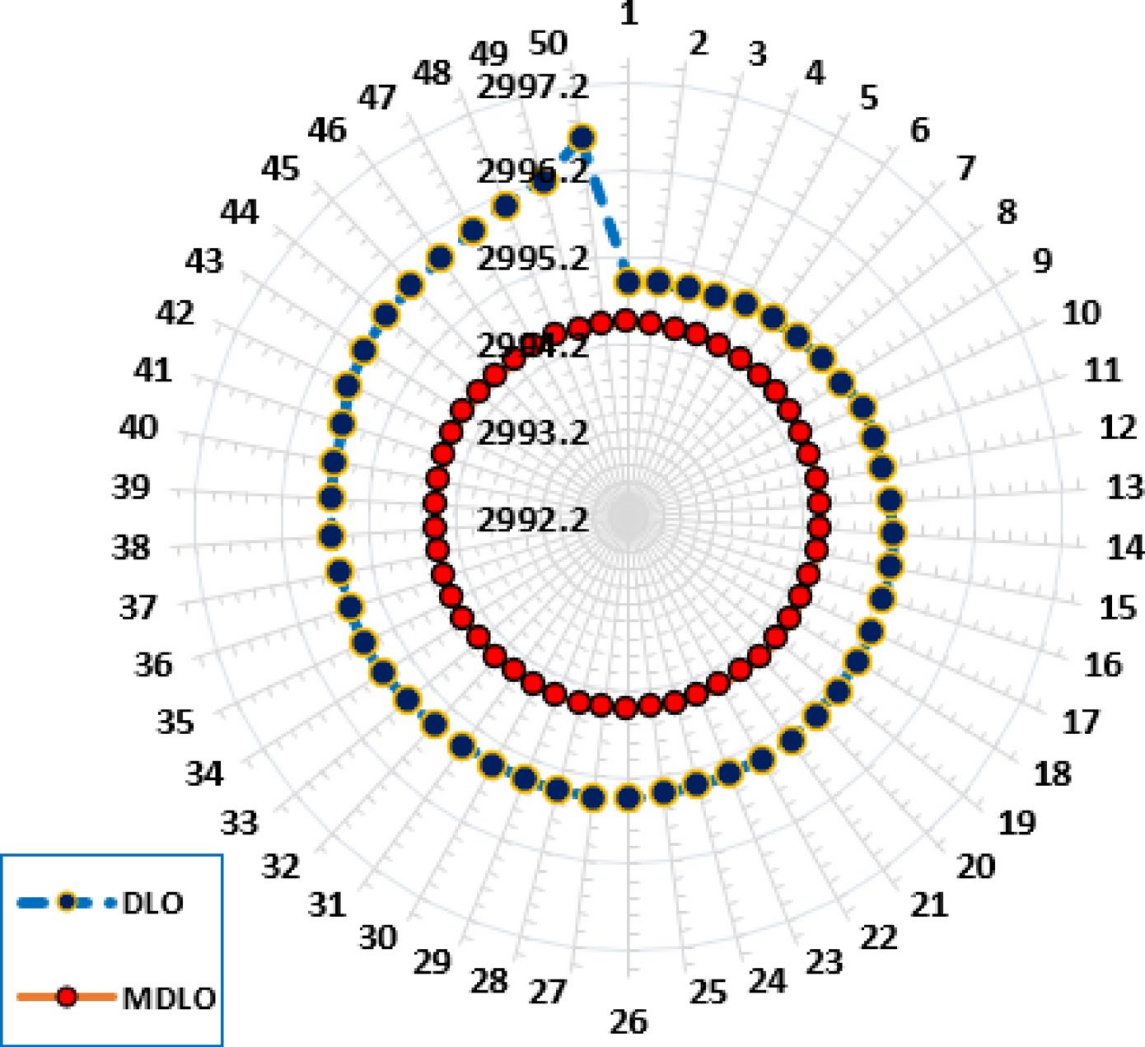
Radar Chart regarding DLO and proposed MDLO for the benchmark model of speed reducer design.

Figure 20 provides a visual comparative representation between DLO and the proposed MDLO. The smaller radial spread associated with MDLO confirms reduced variability and improved convergence accuracy. This graphical comparison reinforces the numerical findings presented in Tables 13, 14 and 15, demonstrating that the proposed modifications significantly enhance stability and precision without introducing performance degradation. The consistent superiority of MDLO across deterministic and statistical measures validates its effectiveness for constrained engineering design optimization problems.

## Conclusions

This paper introduces the MDLO to precisely determine the unidentified components of PEMFC mathematical model since it is complicated and non-linear, making it difficult. This has been addressed by using the voltage–current (V–I) and voltage–power (V–P) features, which are affected by gas pressure as well as operating temperature, as crucial indicator characteristics for parameter determination. The BCS500W, NedStackPS6, and 250 W PEMFC stack references and datasheets are examined in this study. Additionally, the newly created MDLO methodology has been used with a creative modification of the selection operation. Its statistical and numerical effectiveness is being assessed by comparing it with the conventional DLO and alternative optimization methods. Using real experimental data, the convergence properties of MDLO allowed for the optimal adjustment of seven nonlinear characteristics of the PEMFC stack model. Among the different PEMFC cells, the MDLO method provided the lowest sum of squared errors (SSE). In comparison to other optimization techniques, it also recorded the lowest average values of mean error and absolute error (AE). This data emphasizes how well the MDLO technique works to efficiently examine and analyze PEMFC devices.

Moreover, additional statistical and robustness analyses have been incorporated to further strengthen the validity of the findings. Non-parametric Friedman tests, variance and coefficient of variation (CV) analysis, and bootstrap confidence intervals at multiple confidence levels have been conducted to rigorously assess performance stability. Computational complexity, runtime efficiency, and memory utilization have also been evaluated to demonstrate scalability and practical feasibility. Furthermore, cross-validation across three PEMFC stacks with different power ratings has been discussed to confirm generalization capability. These tests provide stronger statistical evidence and reinforce the robustness, reliability, and practical relevance of the proposed MDLO approach.

### Limitations of the study

Although the proposed MDLO demonstrated strong performance and robustness for PEMFC parameter identification, several limitations should be acknowledged. The current study was mainly validated using benchmark PEMFC datasets under steady-state operating conditions, which may not fully represent practical environments involving dynamic temperature, pressure, humidity, and load variations. In addition, the proposed framework focuses on offline parameter extraction rather than real-time online estimation. While MDLO maintains reasonable computational efficiency, practical deployment in embedded controllers or smart-grid energy management systems may require further computational optimization. Moreover, the present work does not include hardware-in-the-loop (HIL) or experimental real-time validation, and therefore practical challenges such as measurement noise, communication delays, and hardware nonlinearities remain unaddressed. Finally, although the proposed method was validated on multiple PEMFC stacks, broader testing on additional industrial-scale systems would further strengthen the generalizability of the proposed framework.

### Future research directions

Future work may focus on extending the proposed MDLO framework toward real-time online PEMFC parameter identification under dynamically varying operating conditions. Hardware-in-the-loop (HIL) implementation and experimental validation using embedded controllers or real PEMFC platforms represent important future directions to evaluate practical applicability and real-time performance. In addition, integrating MDLO with intelligent techniques such as reinforcement learning, digital twins, and predictive control may further improve adaptive capability and robustness. Future studies may also investigate multi-objective optimization formulations considering accuracy, computational cost, efficiency, and degradation simultaneously. Furthermore, uncertainty-aware optimization, parallel computing strategies, and GPU-based acceleration may improve suitability for large-scale smart-grid and hydrogen-energy applications. The proposed MDLO may also be extended to broader engineering optimization problems including renewable-energy management, optimal power flow, and hybrid energy-system optimization.

## Data Availability

The authors declare that the data supporting the findings of this study are available within the paper.

## Appendix A

Tables A1, A2 and A3 tabulate the experimental and simulated voltage, current and power and the associated individual absolute errors between the simulated and experimental voltage and power for the BCS500W, the 250 W and the NedStack PS6 Stacks, respectively.

**Table A1:** The percentage of the individual absolute errors between the simulated and experimental voltage and power of the BCS500W Stack.

Order	Experimental Current	Experimental Voltage	Simulated Voltage	Experimental Power	Simulated Power	Absolute Error in Voltage	Absolute Error in Power	% of VAE	% of PAE
1	0.6	29	28.9972	17.4	17.3983	0.0028	0.0017	0.009655172	0.00977
2	2.1	26.31	26.3059	55.251	55.2425	0.0041	0.0085	0.015583428	0.01538
3	3.58	25.09	25.0936	89.8222	89.8349	0.0036	0.0127	0.014348346	0.01414
4	5.08	24.25	24.2546	123.19	123.2135	0.0046	0.0235	0.018969072	0.01908
5	7.17	23.37	23.3754	167.5629	167.6017	0.0054	0.0388	0.023106547	0.02316
6	9.55	22.57	22.5846	215.5435	215.6831	0.0146	0.1396	0.064687638	0.06477
7	11.35	22.06	22.0713	250.381	250.5096	0.0113	0.1286	0.051223935	0.05136
8	12.54	21.75	21.7585	272.745	272.8511	0.0085	0.1061	0.03908046	0.0389
9	13.73	21.45	21.4613	294.5085	294.6631	0.0113	0.1546	0.052680653	0.05249
10	15.73	21.09	21.0877	331.7457	331.5372	0.0023	0.2085	0.010905642	0.06285
11	17.02	20.68	20.6945	351.9736	352.2205	0.0145	0.2469	0.070116054	0.07015
12	19.11	20.22	20.231	386.4042	386.6141	0.011	0.2099	0.054401583	0.05432
13	21.2	19.76	19.7709	418.912	419.144	0.0109	0.232	0.055161943	0.05538
14	23	19.36	19.366	445.28	445.4186	0.006	0.1386	0.030991736	0.03113
15	25.08	18.86	18.8665	473.0088	473.171	0.0065	0.1622	0.034464475	0.03429
16	27.17	18.27	18.2747	496.3959	496.5242	0.0047	0.1283	0.025725233	0.02585
17	28.06	17.95	17.9533	503.677	503.7699	0.0033	0.0929	0.018384401	0.01844
18	29.26	17.3	17.2929	506.198	505.9896	0.0071	0.2084	0.041040462	0.04117

**Table A2:** The percentage of the individual absolute errors between the simulated and experimental voltage and power of the 250 W Stack.

Order	Experimental Current	Experimental Voltage	Simulated Voltage	Experimental Power	Simulated Power	% of VAE	% of PAE
1	0.5	23.5	23.4793	11.75	11.7397	0.088085106	0.087659574
2	2.1	21.5	21.2561	45.15	44.6378	1.134418605	1.134440753
3	2.8	20.5	20.7645	57.4	58.1405	1.290243902	1.290069686
4	4	19.9	20.1128	79.6	80.4512	1.069346734	1.069346734
5	5.7	19.5	19.3978	111.15	110.5677	0.524102564	0.52388664
6	7.1	19	18.9051	134.9	134.2265	0.499473684	0.49925871
7	8	18.5	18.6161	148	148.9292	0.627567568	0.627837838
8	11.1	17.8	17.7163	197.58	196.6511	0.470224719	0.470138678
9	13.7	17.3	17.0181	237.01	233.1473	1.629479769	1.629762457
10	16.5	16.2	16.2725	267.3	268.4954	0.447530864	0.447212869
11	17.5	15.9	15.9983	278.25	279.97	0.618238994	0.618149146
12	18.9	15.5	15.5972	292.95	294.7878	0.627096774	0.62734255
13	20.3	15.1	15.1582	306.53	307.7121	0.385430464	0.385639252
14	22	14.6	14.4854	321.2	318.6782	0.784931507	0.785118306
15	22.9	13.8	13.8223	316.02	316.5298	0.161594203	0.161318904

**Table A3:** The percentage of the individual absolute errors between the simulated and experimental voltage and power of the NedStack PS6 Stack.

Order	Experimental Current	Experimental Voltage	Simulated Voltage	Experimental Power	Simulated Power	% of VAE	% of PAE
1	2.25	61.64	62.3354	138.69	140.254676	1.12816353	1.128182385
2	6.75	59.57	59.7628	402.0975	403.399069	0.323652845	0.323694971
3	9	58.94	59.0322	530.46	531.289582	0.156430268	0.156389182
4	15.75	57.54	57.4823	906.255	905.345824	0.100278067	0.100322352
5	20.25	56.8	56.7051	1150.2	1148.27925	0.167077465	0.166992452
6	24.75	56.13	56.0334	1389.2175	1386.82668	0.172100481	0.172098431
7	31.5	55.23	55.1486	1739.745	1737.1794	0.147383668	0.147469885
8	36	54.66	54.6135	1967.76	1966.08589	0.08507135	0.085076977
9	45	53.61	53.629	2412.45	2413.30723	0.035441149	0.035533492
10	51.75	52.86	52.9423	2735.505	2739.766	0.155694287	0.155766512
11	67.5	51.91	51.4433	3503.925	3472.42211	0.899056059	0.899074361
12	72	51.22	51.0323	3687.84	3674.32664	0.366458415	0.366430179
13	90	49.66	49.4296	4469.4	4448.6629	0.463954893	0.463979584
14	99	49	48.6413	4851	4815.49278	0.732040816	0.731956617
15	105.8	48.15	48.0474	5094.27	5083.41325	0.213084112	0.213116954
16	110.3	47.52	47.6541	5241.456	5256.25136	0.28219697	0.282275761
17	117	47.1	47.0673	5510.7	5506.87031	0.069426752	0.069495453
18	126	46.48	46.2743	5856.48	5830.55813	0.442555938	0.442618675
19	135	45.66	45.4732	6164.1	6138.88823	0.409110819	0.40900967
20	141.8	44.85	44.861	6359.73	6361.29568	0.024526198	0.0246186
21	150.8	44.24	44.0394	6671.392	6641.14637	0.453435805	0.453363136
22	162	42.45	42.9954	6876.9	6965.25631	1.284805654	1.284827625
23	171	41.66	42.136	7123.86	7205.26318	1.142582813	1.142683531
24	182.3	40.68	41.0269	7415.964	7479.21247	0.852753196	0.852869144
25	189	40.09	40.3514	7577.01	7626.41969	0.652032926	0.652100123
26	195.8	39.51	39.6506	7736.058	7763.58866	0.355859276	0.355874539
27	204.8	38.73	38.697	7931.904	7925.14498	0.085205267	0.085213125
28	211.5	38.15	37.9657	8068.725	8029.75578	0.483093054	0.482966307
29	220.5	37.38	36.9512	8242.29	8147.74502	1.147137507	1.147071723

## Appendix B

The weight of a speed reducer used in small aircraft engines, subject to a set of nonlinear constraints, as defined through the following equations:

Minimize

A.1 $$\begin{gathered} F\left( x \right) = 0.7854x_{1} x_{2}^{2} \left( {14.9334x_{3} + 3.3333x_{3}^{2} - 43.0934} \right) \hfill \\ + 7.4777\left( {x_{6}^{3} + x_{7}^{3} } \right) + 0.7854\left( {x_{4} x_{6}^{2} + x_{5} x_{7}^{2} } \right) - 1.508x_{1} \left( {x_{6}^{2} + x_{7}^{2} } \right) \hfill \\ \end{gathered}$$

Subject to

A.2 $$G_{1} \left( x \right) = 27 - x_{1} x_{3} x_{2}^{2} \le 0$$

A.3 $$G_{2} \left( x \right) = 397.5 - x_{1} x_{2}^{2} x_{3}^{2} \le 0$$

A.4 $$G_{3} \left( x \right) = 1.93 - x_{2} x_{3} x_{4}^{{ - 3}} x_{6}^{4} \le 0$$

A.5 $$G_{4} \left( x \right) = 1.93 - x_{2} x_{3} x_{5}^{{ - 3}} x_{7}^{4} \le 0$$

A.6 $$G_{5} \left( x \right) = \frac{{10}}{{x_{6}^{3} }} \times \left( {16.91 \times 10^{6} + \left( {745\frac{{x_{4} }}{{x_{2} x_{3} }}} \right)^{2} } \right)^{{ - 2}} - 1100 \le 0$$

A.7 $$G_{6} \left( x \right) = \frac{{10}}{{x_{7}^{3} }} \times \left( {157.5 \times 10^{6} + \left( {745\frac{{x_{5} }}{{x_{2} x_{3} }}} \right)^{2} } \right)^{{ - 2}} - 850 \le 0$$

A.8 $$G_{7} \left( x \right) = x_{2} x_{3} - 40 \le 0$$

A.9 $$G_{8} \left( x \right) = 5 - \frac{{x_{1} }}{{x_{2} }} \le 0$$

A.10 $$G_{9} \left( x \right) = \frac{{x_{1} }}{{x_{2} }} - 12 \le 0$$

A.11 $$G_{{10}} \left( x \right) = 1.5x_{6} - x_{4} + 1.9 \le 0$$

A.12 $$G_{{11}} \left( x \right) = 1.1x_{7} - x_{5} + 1.9 \le 0$$

A.13 $$\begin{gathered} 0.7 \le x_{1} \le 0.8,{\text{ }}17 \le x_{2} \le 28,{\text{ }}2.6 \le x_{3} \le 3.6,{\text{ }} \hfill \\ 7.3 \le x_{4} \le 8.3,{\text{ }}7.3 \le x_{5} \le 8.3,{\text{ }}2.9 \le x_{6} \le 3.9,{\text{ }}5 \le x_{7} \le 5.5 \hfill \\ \end{gathered}$$
